# High-density genetic map construction and quantitative trait loci identification for growth traits in (*Taxodium distichum var. distichum* × *T. mucronatum*) × *T. mucronatum*

**DOI:** 10.1186/s12870-018-1493-0

**Published:** 2018-11-01

**Authors:** Ying Yang, Lei Xuan, Chaoguang Yu, Ziyang Wang, Jianhua Xu, Wencai Fan, Jinbo Guo, Yunlong Yin

**Affiliations:** Plant Ecology Research Center, Institute of Botany, Jiangsu Province and Chinese Academy of Sciences, Nanjing, China

**Keywords:** *Taxodium*, Specific locus amplified fragment sequencing, Genetic map, High density, Growth trait, Quantitative trait loci

## Abstract

**Background:**

‘Zhongshanshan’ is the general designation for the superior interspecific hybrid clones of *Taxodium* species, which is widely grown for economic and ecological purposes in southern China. Growth is the priority objective in ‘Zhongshanshan’ tree improvement. A high-density linkage map is vital to efficiently identify key quantitative trait loci (QTLs) that affect growth.

**Results:**

In total, 403.16 Gb of data, containing 2016,336 paired-end reads, was obtained after preprocessing. The average sequencing depth was 28.49 in *T. distichum* var. *distichum*, 25.18 in *T. mucronatum*, and 11.12 in each progeny. In total, 524,662 high-quality SLAFs were detected, of which 249,619 were polymorphic, and 6166 of the polymorphic markers met the requirements for use in constructing a genetic map. The final map harbored 6156 SLAF markers on 11 linkage groups, and was 1137.86 cM in length, with an average distance of 0.18 cM between adjacent markers. Separate QTL analyses of traits in different years by CIM detected 7 QTLs. While combining multiple-year data, 13 QTLs were detected by ICIM. 5 QTLs were repeatedly detected by the two methods, and among them, 3 significant QTLs (*q6–2*, *q4–2* and *q2–1*) were detected in at least two traits. Bioinformatic analysis discoveried a gene annotated as a leucine-rich repeat receptor-like kinase gene within *q4–2*.

**Conclusions:**

This map is the most saturated one constructed in a Taxodiaceae species to date, and would provide useful information for future comparative mapping, genome assembly, and marker-assisted selection.

**Electronic supplementary material:**

The online version of this article (10.1186/s12870-018-1493-0) contains supplementary material, which is available to authorized users.

## Background

The *Taxodium* genus contains extremely flood-tolerant conifer species, including three taxa, i.e. baldcypress (*T. distichum* var. *distichum*), pondcypress (*T. distichum* var. *imbricarium*) and montezuma cypress(*T. mucronatum*). They are diploid, with the chromosome number (n) in every haploid cell11 (2n = 22) [1]. The DNA content of baldcypress’s diploid cell was measured to be 19.90 pg/2C, using flow cytometry [2]. Thus a diploid genome size was about 19.46 Gb, estimated using the formula of Dolezel et al. [3]. *Taxodium* is native to North America and Mexico, and was introduced to China in 1917. Constant cross and backcross breeding efforts have been made among the three *Taxodium* species since the 1970s, and a batch of excellent new varieties named ‘Zhongshanshan’ have been selected from their hybrids. *Taxodium* hybrids ‘Zhongshanshan’ are now widely grown for economic and ecological purposes in southern China, primarily due to their fast growth, straight trunk, good wood properties, and strong adaptability to various environments [4, 5].

Growth traits are the primary determinant of adaptation and productivity for forest tree species. Tree growth is a complex muti-factorial trait determined by the expansion and division of cells in the apical and cambial meristems, photosynthesis efficiency level, water and nutrient use effiency, phenology, and abiotic/biotic stress resistance. Quantitative trait loci (QTL) mapping has remarkable advantages in revealing the genetic architecture of complex traits like growth [6]. A dense genetic map is the determinant for efficient and accurate QTL mapping. Until 2014, the density of forest tree genetic maps were generally low, mostly between 2.7 and 17.6 centiMorgans (cM) [7], mailnly due to the limited number and low polymorphism of the markers used.

Single nucleotide polymorphism (SNP) markers are powerful tools in genetics because of their abundance and even distribution in genomes. Current techniques used for large-scale SNP genotyping of conifers include SNP genotyping chip [8–11], exome capture [12] and restriction-enzyme-based next-generation sequencing (NGS) [13]. One of the most difficult problems in the application of SNP genotyping chip and exome capture technique to non-model species is the acquisition of probes. The design of probes need the availability of high-quality reference genome sequences, which is still difficult to achieve for most conifers. Besides, the development of large-scale probes is usually cumbersome, time-consuming and expensive. Restriction-enzyme-based NGS integrating marker development, sequencing and genotyping into one experimental process [14], has great advantages in efficiency over the others, especially for non-model species with no existing genomic data. The restriction-enzyme-based NGS techniques can be divided into two categories according to whether or not they select fragment sizes prior to PCR amplification [14, 15]. The first category, represented by reduced-representation libraries (RRLs) and restriction-site-associated DNA sequencing (RAD-seq) (and its derivative techniques), conducts the size selection step of the digested fragment before PCR amplification. The other category, represented by genotyping by sequencing(GBS), does not select the size of the digested fragment before PCR amplification. The size selection of digested fragments is very important to improve the efficiency of tag utilization. Compared with GBS technology, RAD technology can obtain more consistent tags among different products under the same amount of sequencing data [15].

The classical RAD-seq technology has several shortcomings, such as more operation steps and shorter read length. Specific-locus amplified fragment sequencing (SLAF-seq) [16] is an improved RAD-seq technology developed in recent years. SLAF-seq uses bioinformatics method to simulate the results of enzyme digestion, selects the most suitable restriction enzymes for double digestion according to research needs, and then carries out double end sequencing on Illumina platform. SLAF-seq technology can effectively avoid repetitive sequences in the genome, increase the effective reads obtained by sequencing, improve the efficiency of molecular marker development, and develop SNP markers with good stability and uniform distribution in the genome. SLAF-seq has been efficient in constructing dense genetic maps with mean marker distances ≤1 cM in many woody plants. For example, the mean marker distance is 1.0 cM in *Camellia sinensis* [17], 0.95 cM in *Juglans regia* [18]**,** 0.82 cM in *Salix matsudana* [19] and 0.774 cM in *Paeonia* Sect. *Moutan* [20]**.** Therefore, SLAF-seq is a mature technology that can be used for large-scale de novo marker development and high-density map construction of woody plants with complex genomes.

For *Taxodium*, only a framework genetic map using sequence-related amplified polymorphisms and SSR markers have been generated to date [21], which produced a low map density with only 179 markers. The total map distance was 976.5 cM, with an average distance of 7.0 cM between markers, and there were34 linkage groups (LGs), which was about triple the haploid number of chromosomes in *Taxodium*. This implies a limitation in the effectiveness for QTL analysis. Here, we used an F_1_ interspecific backcross family of *T. distichum* var. *distichum* × *T. mucronatum* that contained 157 individuals to construct a dense genetic map using SLAF-seq technology. QTLs underlying the growth traits and candidate genes correlated to four growth traits, seedling height (SH), basal diameter (BD), crown width (CW) and diameter at breast height (DBH), were also analyzed. To our knowledge, this is the first high-density genetic map developed in *Taxodium*.

## Methods

### Plant material and DNA isolation

The backcross population of *T. distichum* var. *distichum* and *T. mucronatum* was used as the mapping population. *T. distichum* var. *distichum* is an “elite” clone selected from the arboretum at the Institute of Botany in Nanjing, Jiangsu Province. *T. mucronatum* was planted on the campus of Southeast China University. In 1973, an interspecific hybridization was carried out between *T. distichum* var. *distichum* (female) and *T. mucronatum* (male) at the Institute of Botany. Then an excellent clone named ‘Zhongshanshan 302’ was selected from the F_1_ hydrids in 1988. We bred the interspecific BC_1_ population (*T. distichum* var. *distichum* × *T. mucronatum*) × *T. mucronatum* in 2011 in which the F_1_ ‘Zhongshanshan 302’ was used as the seed parent and the same *T. mucronatum* individual was the recurrent parent. This 5 years old population was planted at a nursery site in Jurong, Jiangsu Province now, and among them, 157 real BC_1_ progenies were used to draw the map. Fresh tender leaves of all the BC_1_ progenies and their parents were collected in Spring, and their genomic DNA were extracted following the protocol of the Plant Genomic DNA Rapid Extraction Kit (Bioteke, Beijing, China).

SLAF-seq library construction, sequencing and SLAF marker development.

SLAF-seq technique [16] was used to develop high-density molecular markers for the 157 BC1 progeny and two parents. When the experiment was started, there was no reference genome released for Taxodiaceae species, so *Picea asperata* Mast (http://congenie.org) was selected for the in silico enzyme digestion prediction based on the *Taxodium* genome size and GC content information. Based on the results of the pre-design experiment, SLAF libraries were constructed as follow. Genomic DNA was first digested with restriction enzyme RsaI (New England Biolabs (NEB), Beverly, MA, USA). Subsequently, an A nucleotide overhang was added to the digested fragments by Klenow Fragment (3′ → 5′ exo−) (New England Biolabs) and dATP at 37 °C. The duplex tag-labeled sequencing adapters (PAGE-purified; Life Technologies, USA) was ligated to the A-tailed fragments by T4 DNA ligase (NEB). Polymerase chain reactions (PCR) were carried out using the diluted restriction–ligation samples, dNTPs, Taq Q5® High-Fidelity DNA Polymerase, the forward primer (5′-AATGATACGGCGACCACCGA-3′), and the reverse primer (5′-CAAGCAGAAGACGGCATACG-3′). The PCR products were then purified using Agencourt AMPure XP beads (Beckman Coulter, High Wycombe, UK) and pooled. The pooled samples were separated using 2% agarose gel electrophoresis. Fragments ranging from 414~ 464 bp (with indexes and adaptors) in size were excised and purified using a QIA quick gel extraction kit (Qiagen, Hilden, Germany). Gel-purified products were then diluted. Paired-end sequencing (125 bp from both ends) was performed using an Illumina HiSeq 2500 system (Illumina, Inc., San Diego, CA, USA) according to the manufacturer’s instructions. *Oryza sativa japonica* was chosen as the control to evaluate the accuracy and validity of the experiment. After sequencing, raw data were filtered, SLAF markers were detected and polymorphic SLAF markers were developed according to the methods of Zhang [19].

### Genetic map construction

The genetic map was constructed using HighMap [22, 23]. Firstly, the recombination rate and modified logarithm of odds (mLOD) scores between SLAF markers were calculated, then the SLAF markers having mLOD scores *<* 3 with all the other SLAF markers were removed, and the remaining SLAF markers were divided into different LGs according to their mLOD scores. Finally, the linear arrangement of markers on each LG and the genetic distances between adjacent markers were obtained used the maximum likelihood method. Kosambi function was used for the mapping. The observed genome length (Go), expected genome length (Ge) and map coverage (Go/Ge) were estimated using methods of Chakravarti et al. [24]. Haplotype maps and marker recombination heat maps were used to evaluate the quality of the map.

### Segregation distortion (SD) analysis

Since distortedly segregated markers are ubiquitous and would affect the mapping results and QTL analysis, partial distorted polymorphism markers showing significance levels between 0.01 and 0.05 (0.01 < *p* < 0.05) were maintained to construct the map. The final number of distorted markers on the map and their distribution on LGs were analyzed. Regions on the map having more than three consecutive adjacent distorted markers were defined as SD regions (SDRs). Since each SLAF marker contained a 100× 2-bp sequence information, they can be used for a deeper analysis. Genes within SDRs were identified through comparisons with the unigene database of ‘Zhongshanshan 406’ [25] and ‘Zhongshanshan 405’ [26] using the BLASTN algorithm. In total, the libraries of ‘Zhongshanshan 406’ [25] and ‘Zhongshanshan 405’ [26] contained 23.1 G and 27.5 Gbyte of clean data and generated 108,692 and 70,312 unigenes, respectively. To guarantee the accuracy of the result, strict thresholds were set with an E-value cut-off of 1e-40 and identities greater than 95% were required.

QTL mapping for growth traits and annotation of genes within major QTL intervals.

The four growth traits were measured in 2–3 continuous years. The SH was measured in three years, 2014–2016, the BD and CW were measured in 2014–2015, and the DBH was measured in 2015 and 2016. The SH, BD and DBH were measured between November and December, and the CW was measured between July and August. The phenotypic measurement of SH, BD and CW were conducted according to the method of Wang et al. [12], and DBH was measured on the main stem, 1.35 m above the ground. The correlation coefficients among all the phenotypes were analyzed by the SPSS 16.0 statistical software.

QTLs underlying the four growth traits were identified by both composite interval mapping (CIM) and inclusive composite interval mapping (ICIM) methods using R/QTL v3.1.1 [27] and IciMapping software v4.0 [28] software, respectively. Firstly, QTLs of each trait in different years were identified by CIM methods with the package R/QTL. The LOD significance thresholds were determined using 1000 permutation test (*P* < 0.05). QTLs with a LOD value between the permutation test LOD threshold and 2.0 were identified as suggestive QTLs. Then, a combined analysis of individual traits in the 2–3 environments were conducted using QTL IciMapping software v4.0. QTL detection was performed using the ICIM method under the bi-parental populations model, and the LOD threshold was set to be 2.5. Genes in the target QTL regions were also predicted using the BLASTN algorithm as mentioned above.

## Results

### Analysis of SLAF-seq data and SLAF marker detection

**Fig. 1:**
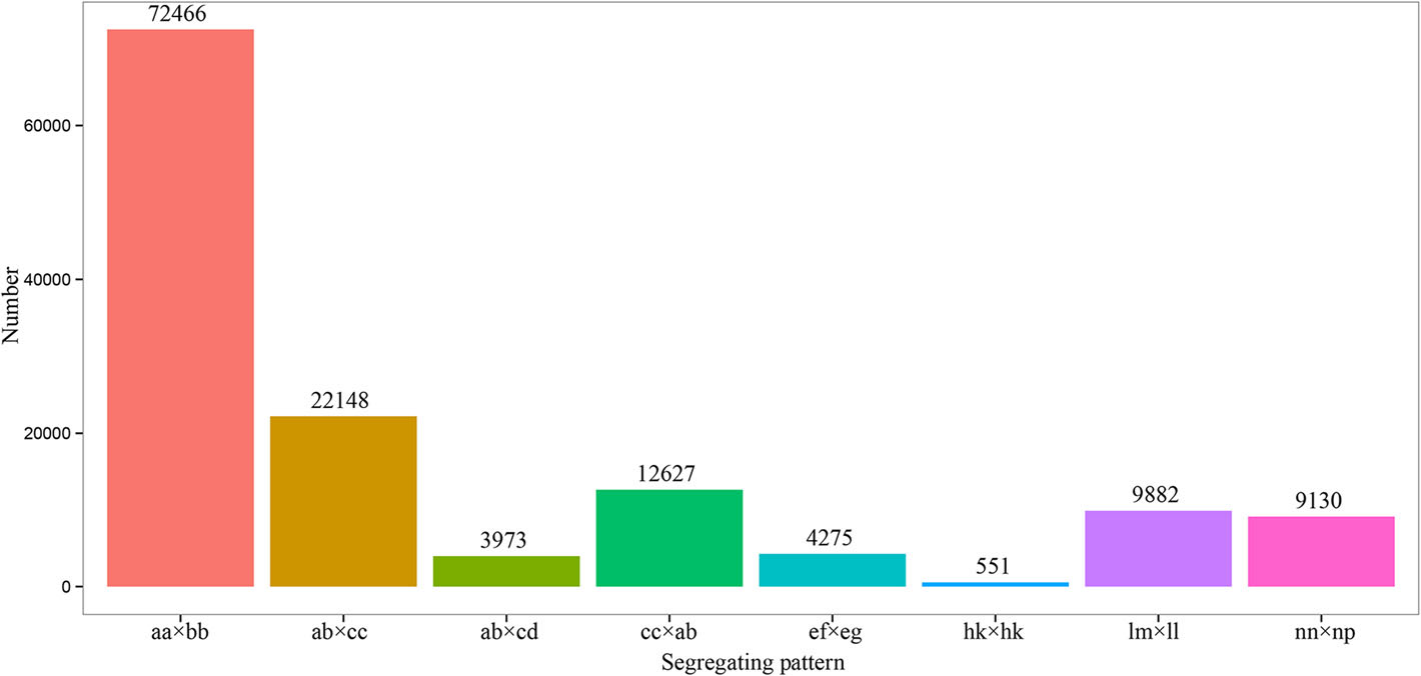
Gnotype distribution of SLAF markers. The Y axis is the number of SLAFs, the X axis is the type of SLAFs

Illumina sequencing data were deposited in the NCBI SRA database under accession number PRJNA486869. 40,766,678 paired-end reads, 30,306,654 paired-end reads, and 12,082,377 paired-end reads were generated for *T. distichum* var. *distichum* (male parent), *T. mucronatum* (female parent), and the BC_1_ individuals, respectively (Table 1). The percentage of bases with a Phred value > 30 was 90.59%, and the average guanine–cytosine content was 36.22%. Based on the high quality paired-end reads, 524,662 SLAFs were defined (Table 1). The average sequencing depths of SLAFs in *T. distichum* var. *distichum* and *T. mucronatum* were 28.49-fold and 25.18-fold, respectively, and the average sequencing depth in BC_1_ plants was 11.12–fold (Table 1). Of these 524,662 SLAFs, 249,619 (47.58%) were polymorphic, and 593 (0.11%) were located in repetitive regions (Table 2). Then, the 249,619 polymorphic SLAFs were sorted into eight segregation patterns as follows: aa×bb, ab×cc, ab×cd, cc × ab, ef × eg, hk × hk, lm × ll and nn × np. Because the BC_1_ population is considered an inbreeding population, only the 72,466 SLAFs falling into the aa×bb segregation pattern could be used for linkage analysis (Fig. 1).

**Table 1:** SLAF-seq data summary

	Female parent	Male parent	Offspring	Total
Total reads				
No. of reads	40,766,678	30,306,654	12,082,377	2016.336(M)
Q30 Percentage(%)	88.32	89.98	90.61	90.59
GC Percentage(%)	36.53	36.41	36.22	36.22
Initial SLAFs				
No. of SLAFs	303,472	337,850	312,008	524,662
Total SLAF depth	8,645,341	8,508,609	3,468,234	
Average SLAF depth	28.49	25.18	11.12	
SLAF markers on the map				
No. of SLAF markers	6156	6156	6126	
Total Depth	347,532	391,400	187,855	
Average Depth	56.45	63.58	30.67	

‘GC’ represents guanine-cytosine, ‘Q30 Percentage’ represents the percentage of bases with a Phred value > 30

**Table 2:** Classification of SLAFs

Type	Polymorphic	Non-polymorphic	Repetitive	Total
Number	249,619	274,450	593	524,662
Percentage	47.58%	52.31%	0.11%	100.00%

### High-density genetic map

**Fig. 2:**
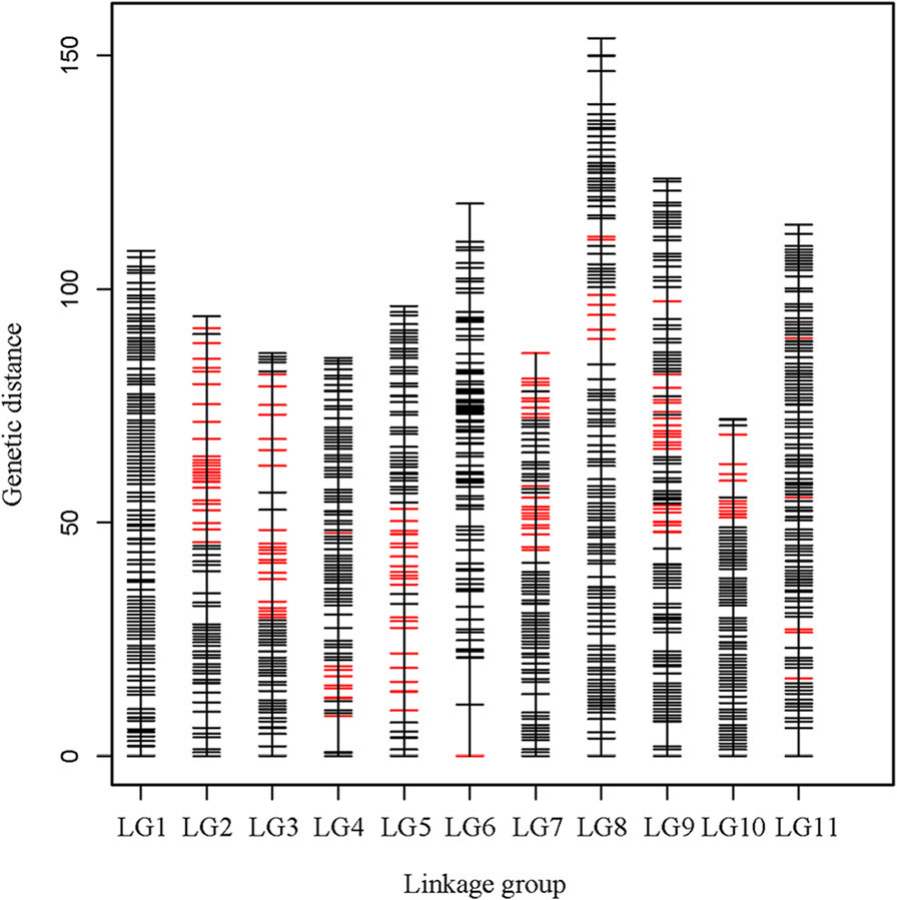
Distribution of SLAF markers of the 11 LGs. A bar indicates a marker and the segregation disrortion markers are highlighted in red. The *x*-axis indicates LG and the *y*-axis represents genetic distance (cM as unit)

To ensure the accuracy of genotyping, the SLAFs that met any of the following criteria were removed: (1) depths of less than 10-fold in each parent; (2) more than three SNPs; (3) a genotype integrity in the BC_1_ population of less than 90%; and (4) significant SD (*p* < 0.01). After filtering, a final set of 6166 high-quality SLAF markers were used for genetic map construction using the HighMap [22] method. As a result, a high-density genetic map harboring 6156 SLAF markers on 11 LGs was constructed, while the remaining10 SLAF markers failed to be linked to any group (Table 3, Fig. 2). The average sequencing depths of these markers were 56.45-fold in *T. distichum* var. *distichum*, 63.58-fold in *T. mucronatum*, and 30.67-fold in BC_1_ individuals (Table 1). The Go were 1137.86 cM, and the average observed map distance between two adjacent assigned markers was 0.18 cM (Table 3). The length of each LG ranged from 86.2 cM (LG7) to 153.72 cM (LG8), and the number of SLAF markers per LG varied from 90 (LG6) to 987 (LG11). The degree of linkage between markers was reflected by a gap ≤5 cM and ranged from 99.45 to 100%, with an average value of 99.79%. The largest gap on the map was 10.91 cM and located in LG6. Because usually one SLAF can harbor 1~ 3 SNPs, the 6156 SLAF markers, harbored 10,710 SNPs, and 64.52% of them were transition-type SNPs (Table 3). Haplotype maps showed that none of the LGs had singleton, expect a low singleton percent (1.74) for LG6. Similarly, none of the LGs had missing SLAFs, expect a 4.71% on LG6. Thus there was a good linear order of markers on LGs (Additional file 1). Heat maps showed that the linkage relation were strong between adjacent markers, and became gradually weaker as the marker distances increasing, indicating the correct order of the markers on LGs (Additional file 2).

**Table 3:** Description on basic characteristics of the 11 LGs

LG ID	SLAF					SNP			
	Number	Total distance (cM)	Average distance(cM)	Gap < 5 cM(%)	Max gap(cM)	Number	Trv	Tri	Trv/Tri
LG1	794	108.2	0.14	100.00	3.06	1355	480	875	0.55
LG2	336	94.15	0.28	100.00	4.77	585	209	376	0.56
LG3	366	86.18	0.24	99.45	5.7	631	223	408	0.55
LG4	528	85.23	0.16	99.81	7.79	947	325	622	0.52
LG5	491	96.33	0.2	99.80	5.49	858	311	547	0.57
LG6	90	118.3	1.31	96.63	10.91	159	57	102	0.56
LG7	519	86.2	0.17	99.81	5.49	887	296	591	0.5
LG8	916	153.72	0.17	99.78	7.07	1582	582	1000	0.58
LG9	486	123.72	0.25	99.79	5.37	835	289	546	0.53
LG10	643	72.02	0.11	99.84	6.18	1156	429	727	0.59
LG11	987	113.81	0.12	99.90	5.87	1715	599	1116	0.54
Total	6156	1137.86	0.18	99.79	10.91	10,710	3800	6910	0.55

‘Gaps≤5’ represents the percentage of gaps in which the distance between two adjacent markers was smaller than 5 cM; ‘LG’ the abbreviation of linkage group; ‘cM’ means centiMorgan; ‘Trv’ represents transversion-type SNP; ‘Tri’ represents transition-type SNP

### Genome length and coverage estimation

The Ge was 1138.23 cM. Based on this estimation, the genome coverage was 99.97%. With an estimated haploid cell DNA content of 9.73 Gb, the physical distance within 1 cM genetic distance was estimated to be 8.55 Mb. Therefore, the estimated physical distance ranged from 0.94 Mb to 11.2 Mb between adjacent SLAF markers, with an average of 1.54 Mb.

### SD analysis

In total, 562 (9.13%) significantly distorted SLAF markers were integrated into the map (Table 4). They were noted in all LGs except for LG1 (Table 4, Fig. 2). The frequencies of SD for markers in LG2 and LG4 were much higher than those of the other LGs at 22.93% and 20.64%, respectively. The lowest frequency of SD for markers was in LG6, which was the smallest LG with only 90 markers, and it had only one distorted marker (1.11%). The percentages of distorted markers for the two largest LGs (LG11 and LG08) were only 2.03% and 1.42%, respectively. All of the distorted markers were skewed toward the heterozygote (Table 4).

**Table 4:** Summary of segregation distortion markers

Linkage group ID	Number	Frequency (%)	Heterozygote
LG1	0	0	0
LG2	77	22.92	77
LG3	35	9.56	35
LG4	109	20.64	109
LG5	79	16.09	79
L G6	1	1.11	1
LG7	102	19.65	102
LG8	13	1.42	13
LG9	91	18.72	91
LG10	35	5.44	35
LG11	20	2.03	20
Total	562	9.13	562

‘Frequency’ is calculated as the number of distorted markers divided by the total number of mapped markers per LG. ‘Heterozygote’ indicated the number of loci exhibiting skewed genotypic frequencies toward heterozygote

Of the 562 distorted markers, 530 showed clustered distributions in 21 SDRs located on 9 LGs, of which 5 LGs (LG2, LG3, LG5, LG8 and LG10) had 2 SDRs, 2 LGs (LG4 and LG7) had 3 SDRs, LG9 had 4 SDRs and LG11 had only 1 SDR (Table 5). Of these, 14 large SDRs with more than adjacent 10 distorted markers, were distributed on each LG. The largest SDR, clustering 63 distorted markers, was located on LG2 within a window starting at 45.724 cM and ending at 88.353 cM. Because the SDRs were believable to contain genes of interest, a BLAST algorithm-based search of the marker sequences (Additional file 3) in SDRs against ‘Zhongshanshan’ unigene database was also conducted. In total, 17 SLAF markers showed significant similarities to unigenes under strict thresholds (Additional file 4), and 11 of them were annotated (Additional file 5).

**Table 5:** Characteristics of the 21 segregation distortion regions

SDR name	LG	Marker interval	Marker number	Physical distance interval(cM)	Length(cM)
SDR2.1	2	Marker28309-Marker83390	63	45.724–88.353	42.629
SDR2.2	2	Marker14643-Marker93841	14	91.583–91.583	0
SDR3.1	3	Marker50458-Marker36964	28	29.629–48.392	18.763
SDR3.2	3	Marker66278-Marker64302	7	62.193–81.704	19.511
SDR4.1	4	Marker100041-Marker9947	86	8.434–8.434	0
SDR4.2	4	Marker102672-Marker8525	17	12.364–19.106	6.742
SDR4.3	4	Marker2244-Marker88716	6	47.72–47.72	0
SDR5.1	5	Marker110686-Marker42288	14	9.681–29.606	19.925
SDR5.2	5	Marker63315-Marker44926	65	36.697–52.937	16.24
SDR7.1	7	Marker38510-Marker79086	63	44.061–55.321	11.26
SDR7.2	7	Marker108601-Marker63468	8	57.747–57.747	0
SDR7.3	7	Marker45883-Marker27197	8	72.45–76.566	4.116
SDR8.1	8	Marker24306-Marker75217	6	89.299–98.714	9.415
SDR8.2	8	Marker16874-Marker28355	7	110.605–111.181	0.576
SDR9.1	9	Marker33824-Marker97312	28	47.972–53.63	5.658
SDR9.2	9	Marker188850-Marker64057	24	65.755–72.294	6.539
SDR9.3	9	Marker23238-Marker36508	14	73.662–76.255	2.593
SDR9.4	9	Marker125877-Marker76947	23	78.858–81.654	2.796
SDR10.1	10	Marker97780-Marker23964	9	51.069–54.65	3.581
SDR10.2	10	Marker49464-Marker96618	26	58.952–68.754	9.802
SDR11	11	Marker11045-Marker72403	14	55.337–55.337	0

‘SDR’ indicated segregation distortion region

### QTL mapping and candidate gene prediction

**Fig. 3:**
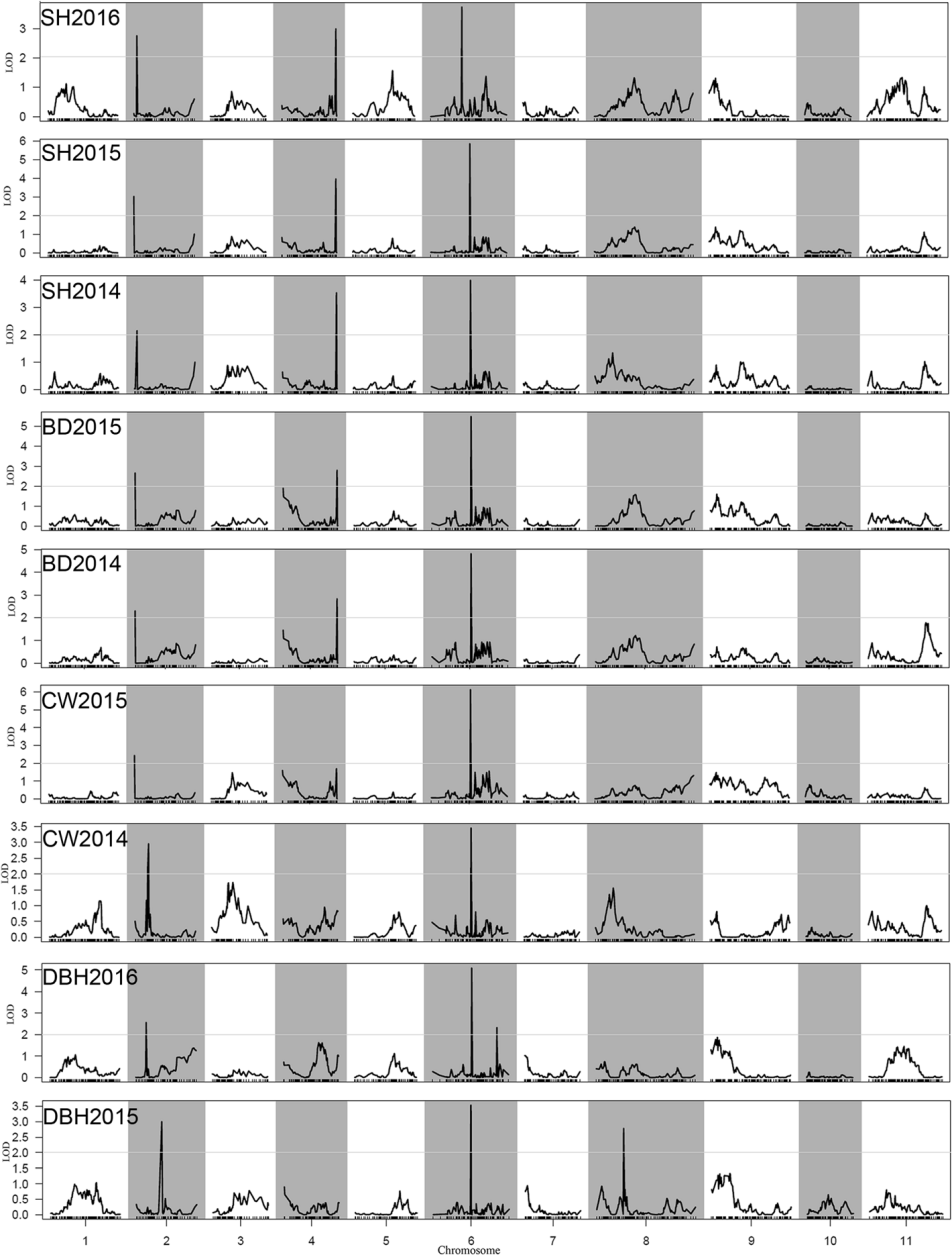
Separate QTL analysis of the 4 growth traits in different years using the CIM method of R/QTL package. The x-axis indicates map position (cM) across the 11 LGs, while the y-axis represents the LOD scores. Horizontal line on the chart represents LOD threshold. ‘SH2016’, ‘SH2015’, and ‘SH2014’ represents seedling height in 2016, 2015 and 2014, respectively. ‘BD2015’ and ‘BD2014’ represents basal diameter in 2015 and 2014, respectively. ‘CW2015’ and ‘CW2014’ represents crown width in 2015 and 2014, respectively. ‘DBH 2016’ and ‘DBH 2015’ represents diameter at breast height in 2016 and 2015, respectively

Phenotypic correlations estimated between any two of the four traits in any particular year were significant (Table 6). Additionally, every trait was highly correlated from year to year, indicating that they were poorly mediated by the growth environment. Separate QTL analyses of the four traits in different years were conducted using the CIM method of R/QTL package, and major QTLs were detected on LG2, LG4, LG6 and LG8 (Fig. 3), with LOD values varying between 2.14 and 6.0 and the proportions of variance explained by each QTL varying between 1.82 and 13.15% (Additional file 6). A genome scan showed that SH2016, SH2015, SH2014, BD2015 and BD2014 exhibited quite similar LOD profiles. Among them, three QTLs were significant. The 3rd significant QTL was detected on the top of LG2, the 2nd was detected on the bottom of LG4 and the 1st was detected in the middle of LG6. In this study, overlapping QTLs or adjacent QTLs separated by less than 5 cM were classified into the same locus. Based on this rule, all of the identified QTLs were classified into seven genomic regions (loci), with three loci on LG2, one on LG4, two on LG6 and one on LG8 (Additional file 6). Among them, the most repeated loci, *q6–2*, *q2–1* and *q4–1*, were detected eight, six and five times across traits and years, and they explained 4.52–13.35% of the observed phenotypic variation. *q2–2* and *q6–1* were detected twice. The remaining two loci, *q2–3* and *q8–1*, were detected only once. In conclusion, separate QTL analyses identified three stable loci, *q6–2*, *q2–1* and *q4–1*, which could all be detected at least five times across traits or years, and were defined as the major QTLs. In particular, *q6–2* had an overlapping peak for all four growth traits and the highest LOD score. Thus, it may play a major role in juvenile growth.

**Table 6:** Year on year correlation for SH, DBH, BD and CW

	SH2014	SH2015	SH2016	DBH2015	DBH2016	BD2014	BD2015	CW2014
SH2015	0.926^**^							
SH2016	0.845^**^	0.953^**^						
DBH2015	0.820^**^	0.944^**^	0.912^**^					
DBH2016	0.727^**^	0.909^**^	0.951^**^	0.945^**^				
BD2014	0.877^**^	0.860^**^	0.822^**^	0.818^**^	0.789^**^			
BD2015	0.853^**^	0.935^**^	0.936^**^	0.935^**^	0.939^**^	0.908^**^		
CW2014	0.844^**^	0.803^**^	0.771^**^	0.781^**^	0.730^**^	0.871^**^	0.822^**^	
CW2015	0.714^**^	0.808^**^	0.810^**^	0.824^**^	0.846^**^	0.813^**^	0.859^**^	0.775^**^

**Correlation is significant at the 0.01 level (2-tailed).‘SH2016’ represents seedling height in 2016, ‘SH2015’ represents seedling height in 2015, ‘SH2014’ represents seedling height in 2014, ‘BD2015’ represents basal diameter in 2015, ‘BD2014’ represents basal diameter in 2014, ‘CW2015’ represents crown width in 2015, ‘CW2014’ represents crown width in 2014, ‘DBH2016’ represents diameter at breast height in 2016, ‘DBH2015’ represents diameter at breast height in 2015

**Fig. 4:**
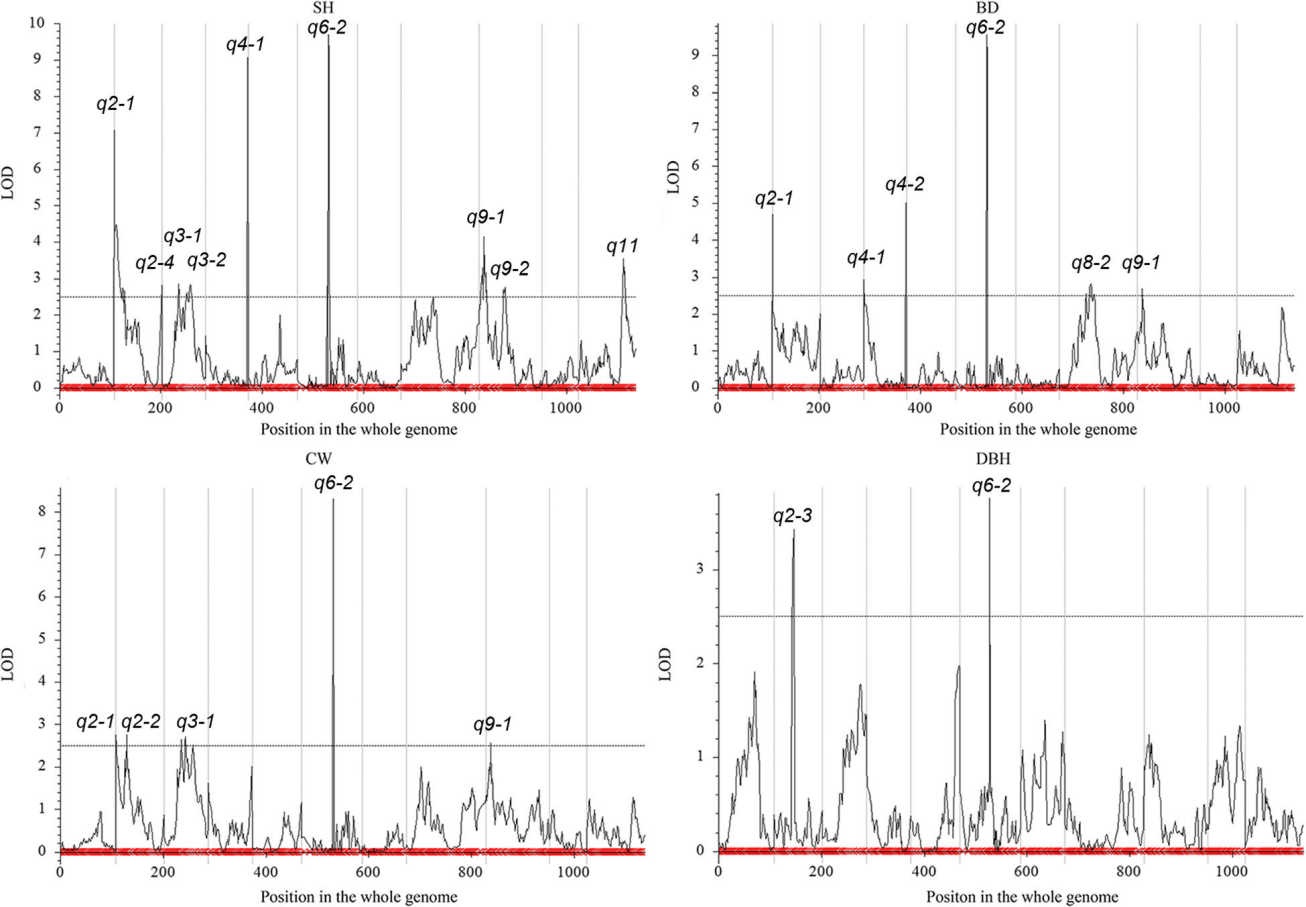
Combined QTL analysis of the 4 growth traits in different years using the ICIM method of IciMapping software. The x-axis indicates map position (cM) across the 11 LGs and the y-axis is LOD score. ‘SH’ represents seedling height. ‘BD’ represents basal diameter. ‘CW’ represents crown width. ‘DBH’ represents diameter at breast height

The combined QTL analyses of individual growth trait in different years was conducted using the ICIM method of IciMapping software. QTLs were identified on six LGs, LG2, LG3, LG4, LG6, LG8, LG9 and LG11. The SH and BD also had the most similar LOD profiles (Fig. 4). In total, 24 QTLs, 10, 6, 5 and 3 QTLs for SH, BD, CW and DBH, respectively, were detected, with LOD scores varying between 2.56 to 9.69, and the PVE (phenotypic variance explained) of each QTL varied from 0.92 to 14.88% (Additional file 7). The 24 QTLs were further classified into 13 genomic loci (Additional file 7) based on the rules mentioned above, among them 5 loci (bold in Additional file 7), *q2–1*, *q2–2*, *q2–3*, *q4–1*and *q6–2* were also identified using the CIM method as mentioned above(Additional file 6). Thus, the ICIM method detected more QTL peaks than the CIM method even though a higher LOD threshold was set. Three loci, *q2–1*, *q4–1* and *q6–2*, were detected as effecting at least two traits (Additional file 7), with *q6–2* effecting four traits, *q2–1* effecting three traits and *q4–1* effecting two traits, and they explained 4.81–14.88% of the observed phenotypic variation.

Comparing the QTL results of CIM and ICIM, the three loci (*q2–1*, *q4–1* and *q6–2*) repeatedly detected in at least two traits by both methods were accepted as stable and reliable QTLs. None of them had overlapping regions with SDRs on the map. Marker intervals within the three QTL confidence intervals identified by either of the two methods were used for further analysis. In total, *q6–2* contained 5 SLAF markers, *q4–1* contained 45 SLAF markers and *q2–1* contained 54 SLAF markers (Additional file 8). A BLAST algorithm-based search of SLAF markers against the unigene database of ‘Zhongshanshan’ found putative-related genes within the three stable QTL regions. Strict thresholds were set to ensure the credibility of the results. For *q6–2*, no annotated genes were predicted because of the scarcity of markers. For *q4–1*, Marker 20,554 showed a significant similarity to unigene comp127245_c0, which was annotated as encoding a receptor-like protein kinase (RLK) (Additional files 9 and 10), and Marker 39,317 had a significant similarity to CL4975Contig1. For *q2–1*, Marker 29,918 showed a significant similarity to comp112880_c1. Neither did CL4975Contig1 nor comp112880_c1 had been annotated.

## Discussion

### SLAF-seq and marker development

The sequencing depth of SLAFs is an important indicator of the sequencing quality. For our raw data, the sequence depth was 11.12× for progeny and greater than 25× for parents. The greater read depths of these markers for parents and progeny ensured sequence and genotyping corrections of SLAFs mapped in the linkage map. We analyzed the sequencing dataset and found that the guanine–cytosine content was 36.22% on average, which is lower than in the sequenced transcriptomes of ‘Zhongshanshan’ [25, 26], which was about 44%. This showed the different characteristics between genomic and transcriptome sequence of *Taxodium*. Similar results were observed with Tree Peony [20]. The statistics of the mapped SNP types showed that 64.52% were transition-type SNPs. This ratio is close to those of most other species, such as 68.23% in *Juglans regia* [18] and *Gossypium hirsutum* [29], 67.72% in *Paeonia* Sect. *Moutan* [20] and 62.44% in *Sesamum indicu* [30] . Of the initial SLAFs, 47.58% were polymorphic between two parents. The polymorphism rate of SLAFs was greater than that reported for the expressed sequence tag–SSR markers (31.52%) in *Taxodium* [31]. The high polymorphic rate demonstrated that the genetic diversity in *Taxodium* is high. Thus, the SLAF-seq results revealed various genetic and genomic information of *Taxodium*.

### Genetic map analysis

Large genome sizes, long generation times, outbred mating systems and the lack of a reference genome made it difficult to construct high-density genetic maps for conifers. We established a segregating population by backcrossing, which was more suitable for genetic map construction than F_1_ population usually used for forest trees. The final map contained 6156 SLAF markers that including10,710 SNPs. The number of LGs obtained was consistent with the haploid cell chromosome number. The Go was 1137.86 cM, and the average distance between markers was only 0.18 cM. Other SNP genotyping techniques have also been successfully applied to construct high-density genetic maps with mean marker distances below 1 cM for confiers, including *Picea glauca* [8]*,Cryptomeria japonica* [9]*, Picea abies* [10]*, Pinus pinaster* [11]*, Pinus taeda* [12]*, and Callitris glaucophylla* [13]. However, compared with those works, the number of mapped markers and marker density are both higher in this map (Table 7), suggesting the high efficiency of SLAF-seq technology in de novo marker development and genotyping of conifers. Besides, since each SLAF markers contained a 100× 2-bp sequence information, our high-density genetic map might be helpful for future comparative genomic analysis and genome assembly of *Taxodium* and other Taxodiaceae species.

**Table 7:** Comparison of the linkage map constructed in this work to previously constructed for some other conifers

Family	Genus	Species	Sample size	No. of mapped markers	Marker type	Genotyping method	No. of linkage groups	Observed map length in cM	Average diatance between markers(cM)	Reference
Taxodiaceae	*Cryptomeria*	*Cryptomeria japonica*	147	2560	Genotyping chip	SNP, SSR, EST-SSR, ALP	11	1266.2	0.49	[9]
	*Taxodium*	*T.* ‘Zhongshanshan 302’ × *T. mucronatum*	148	179	Electrophoretic separation	SRAP, SSR	34	976.5	7.0	[21]
		*T. distichum* var. *distichum* × *T. mucronatum*	157	10,710	SLAF-seq	SNP	11	1137.86	0.18	This study
Pinaceae	*Spruce*	*Picea glauca*	1976	8852	Genotyping chip	SLAF-SNP	12	1895	0.28	[8]
		*Picea abies*	247	686	Genotyping chips	SNP	12	1889.2	0.97	[10]
	*Pinus*	*Pinus taeda*	72	2841	Sequence capture	SNP, indel, MNP	12	1637.4	0.58	[12]
		*Pinus pinaster*		1131	Genotyping chips	SNP	12	1708	0.67	[11]
Cupressaceae	*Callitris*	*Callitris glaucophylla*	88	4284	RAD-seq	SNP, SSR	11	1033.5 M	0.24	[13]

*MNP*, multiple-nucleotide polymorphism

Even though the average distance between markers is as small as 0.18 cM, 0.21% of marker interval spaces were greater than 5 cM and the average distance of the different LGs varied from 0.11 cM (LG10) to 1.32 cM (LG6). Regions of high marker density may occur in centromeric regions with reduced recombination rates, while regions of low marker density are associated with telomeres with higher rates of recombination. Enlarging the mapping population size(*N*) would be an effective way to further improve the resolution of the map. Since the homologous recombination rate of chromosomes during meiosis determines the genetic distance of markers on the map, a larger *N* can ensure the detection of smaller recombination rate, resulting a map with higher density and accurancy.

According to the Ge and estimated genome size, the physical distance within 1 cM genetic distance was estimated to be 8.55 Mb, which is much greater than the estimated 598.98 Kb in *Litopenaeus vannamei* [32]*.* The genetic length is out of proportion to the physical distance, and the number of bases per cM varies greatly in different species. The chemical complexity of the genome directly affects the genetic length, and species with large amounts of genomic DNA, while shorter genetic lengths generally indicate a higher proportion of repetitive elements. Conifer genomes are reputed to be highly repetitive, for example, under permissive conditions, 80% of the *Pinus taeda* genome is repetitive [33]. *Taxodium* has a closer phylogenetic relationship to *Cryptomeria* in the Taxodiaceae family [34] and the genome size of *T. distichum* var. *distichum* (9.95 pg/C) is close to that of *Cryptomeria japonica* (10.05 pg/C) [2]. The estimated haploid cell’s DNA content in *C. japonica* is 10.8 Gb, and the Ge is 1278.5 cM [9], meaning 8.45 Mb base per cM. This is close to the value in *Taxodium*. The observed genome coverages were 99.97%. Therefore, the genetic map has basically reached saturation.

### SD analysis

SD is a common and is thought to be a powerful evolutionary force for plants, since the SD markers always linked to fitness genes. To avoid losing information of such important markers, the 562 SLAF markers that showed significant SD with a *p* value between 0.01 and 0.05 were also inserted in this map. In total, 94.3% of the segregation distorted markers were clustered in 21 SDRs. Gametic selection (male gametic competition and female gametic selection) and zygotic selection play important roles in SDRs [9, 35, 36]. The 21 SDRs in our study may be caused by female meiotic drive or zygotic selection because, in a BC_1_ population in which the F_1_ hybrid was the seed parent, male gametic-specific mechanisms can be excluded for SD [37]. Studies on more reciprocal backcross and F_2_ populations could distinguish the roles of female and zygotic mechanisms in these SDRs [37, 38]. Molecular markers within the same SDR primarily skewed in the same direction, which was heterozygotes in our BC_1_ population (*T. distichum* var. *distichum* × *T. mucronatum*) × *T. mucronatum*, suggesting that heterozygotes were preferred under competition. If female gametic competition was the main factor influencing an SDR, then female gametes containing the *T. distichum* var. *distichum* alleles were preferentially transmitted.

However, if an SDR resulted from zygotic selection, multilocus epistasis, competitive interactions between zygotes or inbreeding depression may be the genetic mechanisms [37]. In outbred forest tree species, inbreeding depression has often been advocated as a critical mechanism affecting zygote viability [39, 40] because trees always have a large genetic load and accumulate large numbers of recessive deleterious mutations in fitness genes, in which homozygosity would decrease the viability of plants. The phenomenon that SD are closely linked with recessive embryonic lethal [39, 41, 42] or deleterious resistance-related genes [43, 44] has been extensively reported in forest trees. In the present study, F_1_ progeny was backcrossed with its male parent *T. mucronatum*. Predictably, many recessive deleterious genes from *T. mucronatum* would become homozygous and cause individual death, as a result, markers linked with these genes tend to skew towards heterozygotes. In our mapping population, all of the mapped distorted markers were skewed towards heterozygotes, inbreeding depression may be the primary mechanism. Blast analysis found that marker40499 in SDR9.1 may be a fragment of a ‘Zhongshanshan’ unigene annotated as a member of the Toll/interleukin-1 receptor-nucleotide binding site-leucine rich repeat (TIR-NBS-LRR) gene family, one of the largest plant disease resistance gene family [45, 46], which may further support this hypothesis.

### Phenotypic correlation analysis

GD and DBH of trees are determined by the secondary growth of vascular cambium. SH is mainly influenced by the primary growth of the shoot apical meristem (SAM). CW is determined by the length and angle of the branches, thus, SAM is also a major factor affecting CW. Researches found that SAM and vascular cambium share overlapping regulatory systems, and most essential SAM regulators are also expressed in the vascular cambium during woody growth, such as members of Class I KNOX transcription factors, homeobox genes, *CLAVATA* genes, Class III homeodomain-leucine zipper transcription factors, KANADI transcription factors [6, 47–49]. Here, SH, GD, DBH and CW had high level of phenotypic correlations, this may be the result of the similar basal regulatory mechanisms underlying primary and secondary meristem of trees.

### QTL analysis of growth traits

The accuracy of QTL mapping is influenced by the mapping method used, and an improper method may lead to erroneous or false positive results. CIM and ICIM methods were both used to ensure the reliability of the identified QTLs. ICIM detected about twice the number of QTLs identified by CIM, indicating that ICIM had higher detection power, which is in agreement with the characteristics of this method [50]. The SH and BD had the most similar LOD profiles in both methods, indicating the strong genetic relevance of the transverse and longitudinal growth of trunk. The PVE of each QTL was, in general, low, varying between 1.82 and 13.15% in the CIM method and between 0.92 and 14.88% in the ICIM method. However, this result is in agreement with previous findings obtained for growth traits in forest trees, with most values being between 4 and 12% [7].Here, the QTLs detected by two methods simultaneously were believed to be highly reliable, and three major QTLs (*q6–2*, *q4–1* and *q2–1*) were found to be stable. In particular, *q6–2* was most frequently detected in both methods with high LOD and PVE scores. This represents a major QTL underlying growth. That QTLs underlying different traits were detected in overlapping regions can explained in three ways: (1) the presence of pleiotropic effects in which a gene has an impact on the metabolic pathways of multiple traits (2) QTLs affecting different traits exist in clusters; and (3) the distances between two adjacent markers are large and may contain multiple QTLs. The markers within the three stable QTLs may have the potential to be used in MAS in ‘Zhongshanshan’ growth breeding. Notably, a growth QTL may sometimes identify genetic loci contributing to inbreeding depression [51], which makes the use of markers within QTLs for MAS more complex. However, in this study, none of the three stable QTLs overlapped with SDRs in the map.

The regions of the three stable QTLs may harbor strong candidate genes for ‘Zhongshanshan’ juvenile growth. The fast development of genome information (such as transcriptomes) with new genotyping technologies has allowed the successful identification of strong candidate genes within QTL intervals in several plants [52, 53]. Three markers showed significant similarities to unigenes, among which, one unigene was annotated as an *LRR-RLK* gene. Plant *LRR-RLK*s are structurally similar to the animal growth factor receptors [54], many of them are specially expressed in vascular tissues and vital for plants’ stem growth [55–57]. Thus, this gene may also play an important role in ‘Zhongshanshan’ secondary vascular system development. The remaining two unigenes with no functional annotations may be new genes, whose function deserve deeper analysis.

The population used for QTL detection here consisted only 157 offsprings, which was relatively small compared to recent work in other conifers [7, 8]. The detection efficiency of QTL may be reduced due to the small *N*. Firstly, *N* affects the accurate positioning of QTL on the map. The bias of the recombination rate estimator decreases with the increasing of *N*, resulting a more accurate calculated distance between QTL and markers [58]. Secondly, N affects the number of signficant QTLs detected, since, LOD increased with the increasing of N [58]. Li et al.[59]inferred that for *N* = 100, 200, and 400, the probability of locating QTL with PVE of 4% to the 10 cM confidence interval was 29%, 67% and 91%, respectively. Thirdly, *N* affects the accurancy of PVE. Xu [58] found that the bias of QTL genetic variance decreased with the increase of *N*. Since PVE is determined by the genetic variance and phenotypic variance of QTL [59], increasing *N* would lead to a downward bias in the estimation of PVE. Therefore, building a much larger population as soon as possible will provide a guarantee for more efficient QTL positioning.

## Conclusion

We constructed a high-density genetic map of *T. distichum* var. *distichum* × *T. mucronatum* using the SLAF-seq strategy and identified three stable major QTLs underlying growth traits that explained the high-phenotypic variation. This laid the foundation for MAS in ‘Zhongshanshan’ for growth breeding. Additionally, with more genome information becoming available for Taxodiaceae using NGS, the identification of more key genes within QTL intervals using the sequence information of SLAF markers may be possible. The candidate gene within the QTL annotated as encoding LRR-RLK protein may be a promising target gene for increasing growth in *Taxodium*, which worth next-step functional verification.

## Additional files

Additional file 2: Heat maps of the genetic map. Tif. Each cell represents the recombination rate of two markers. Yellow indicates a lower recombination rate and purple a higher one. (TIF 15423 kb)

Additional file 3: The 530 SLAF markers encompassed in the 21 SDRs. (XLSX 66 kb)

Additional file 4: Result of the blast analysis of the markers within the SDRs against the unigene database of ‘Zhongshanshan’. (PDF 186 kb)

Additional file 5: The annotation of unigenes showing significant similarities to the SLAF markers within SDRs. (XLSX 16 kb)

Additional file 6: The characters of 7 consensus loci associated with growth-related traits across years detected by the CIM method. (DOC 215 kb)

Additional file 7: The characters of 13 consensus loci associated with growth-related traits across various years detected by the ICIM method. (DOC 60 kb)

Additional file 8: The SLAF markers encompassed in the major growth associated locus as revealed by QTL analysis. (XLSX 24 kb)

Additional file 9: Result of the blast analysis of the markers within the intervals of the three stable QTLs against the unigene database of ‘Zhongshanshan’. (PDF 126 kb)

Additional file 10: The sequence and annotation of unigenes showing significant similarities to growth-linked SLAF markers of ‘Zhongshanshan’. (XLSX 16 kb)

## Abbreviations

BD: Basal diameter
CIM: Composite interval mapping
cM: centiMorgan
CW: Crown width
DBH: Diameter at breast height
GBS: Genotyping by sequencing
Ge: Expected genome length
Go: Observed genome length
ICIM: Inclusive composite interval mapping
LG: Linkage group
LOD: Logarithm of odds
MAS: Marker-assisted selection
NGS: Next-generation sequencing
PVE: Phenotypic variance explained
QTL: Quantitative trait loci
RAD-seq: Restriction-site-associated DNA sequencing
SAM: Shoot apical meristem
SD: Segregation distortion
SDR: Segregation distortion region
SH: Seedling height
SLAF-seq: Specific locus amplified fragment sequencing
SNP: Single nucleotide polymorphism

## References

[1] Coker WC (1903). On the gametophytes and embryo of *Taxodium*. Bot Gaz.

[2] Hizume M, Kondo T, Shibata F, Ishizuka R (2001). Flow cytometric determination of genome size in the Taxodiaceae, Cupressaceae *sensu stricto* and Sciadopityaceae. Cytologia.

[3] Dolezel J, Bartos J, Voglmayr H, Greilhuber J (2003). Nuclear DNA content and genome size of trout and human. Cytometry A.

[4] Creech D, Yin YL (2006). Improvement, propagation and use of *Taxodium* in southeastern China. Chem Soc Rev.

[5] Creech D, Eguiluz-Piedra T (2011). Can Taxodium be improved?. Piedra.

[6] Grattapaglia D, Plomion C, Kirst M, Sederoff RR (2009). Genomics of growth traits in forest trees. Curr Opin Plant Biol.

[7] Muranty H, Jorge V, Bastien C, Lepoittevin C, Bouffier L, Sanchez L (2014). Potential for marker-assisted selection for forest tree breeding: lessons from 20 years of MAS in crops. Tree Genet Genomes.

[8] Pavy N, Lamothe M, Pelgas B, Gagnon F, Birol I, Bohlmann J, Mackay J, Isabel N, Bousquet J (2017). A high-resolution reference genetic map positioning 8.8 K genes for the conifer white spruce: structural genomics implications and correspondence with physical distance. Plant J.

[9] Moriguchi Y, Uchiyama K, Ueno S, Ujino-Ihara T, Matsumoto A, Iwai J, Miyajima D, Saito M, Sato M, Tsumura Y (2016). A high-density linkage map with 2560 markers and its application for the localization of the male-sterile genes *ms3* and *ms4* in *Cryptomeria japonica* D. Don. Tree Genet Genomes.

[10] Lind M, Källman T, Chen J, Ma X, Bousquet J, Morgante M, Zaina G, Karlsson B, Elfstrand M, Lascoux M (2014). A *Picea abies* linkage map based on SNP markers identifies QTLs for four aspects of resistance to *Heterobasidion parviporum* infection. PLoS One.

[11] Chancerel E, Lamy J, Lesur I, Noirot C, Klopp C, Ehrenmann FO, Boury C, Provost GL, Label P, Lalanne C (2013). High-density linkage mapping in a pine tree reveals a genomic region associated with inbreeding depression and provides clues to the extent and distribution of meiotic recombination. BMC Biol.

[12] Neves LG, Davis JM, Barbazuk WB, Kirst M (2014). A high-density gene map of loblolly pine (*Pinus taeda* L.) based on exome sequence capture genotyping. *G3*. Genes Genomes Genetics.

[13] Sakaguchi S, Sugino T, Tsumura Y, Ito M, Crisp MD, Bowman DMJS, Nagano AJ, Honjo MN, Yasugi M, Kudoh H *et al*: High-throughput linkage mapping of Australian white cypress pine (*Callitris glaucophylla*) and map transferability to related species. *Tree Genetics & Genomes*2015, 11(6).

[14] Davey JW, Hohenlohe PA, Etter PD, Boone JQ, Catchen JM, Blaxter ML (2011). Genome-wide genetic marker discovery and genotyping using next-generation sequencing. Nat Rev Genet.

[15] Andrews KR, Good JM, Miller MR, Luikart G, Hohenlohe PA (2016). Harnessing the power of RAD-seq for ecological and evolutionary genomics. Nat Rev Genet.

[16] Sun X, Liu D, Zhang X, Li W, Liu H, Hong W, Jiang C, Guan N, Ma C, Zeng H (2013). SLAF-seq: an efficient method of large-scale de novo SNP discovery and genotyping using high-throughput sequencing. PLoS One.

[17] Ma J, Huang L, Ma C, Jin J, Li C, Wang R, Zheng H, Yao M, Chen L (2015). Large-scale SNP discovery and genotyping for constructing a high-density genetic map of tea plant using specific-locus amplified fragment sequencing (SLAF-seq). PLoS One.

[18] Zhu Y, Yin Y, Yang K, Li J, Sang Y, Huang L, Fan S (2015). Construction of a high-density genetic map using specific length amplified fragment markers and identification of a quantitative trait locus for anthracnose resistance in walnut (*Juglans regia* L.). BMC Genomics.

[19] Zhang J, Yuan H, Li M, Li Y, Wang Y, Ma X, Zhang Y, Tan F (2016). Wu R: a high-density genetic map of tetraploid *Salix matsudana* using specific length amplified fragment sequencing (SLAF-seq). PLoS One.

[20] Cai C, Cheng F, Wu J, Zhong Y, Liu G (2015). The first high-density genetic map construction in tree Peony (*Paeonia* sect. *Moutan)* using genotyping by specific-locus amplified fragment sequencing. PLoS One.

[21] Wang Z, Cheng Y, Yin Y, Yu C, Ying Y, Qin S, Hao Z, Li H (2016). Genetic linkage map construction and QTL mapping of seedling height, basal diameter and crown width of *Taxodium* 'Zhongshanshan 302′ × *T. mucronatum*. Springerplus.

[22] Liu D, Ma C, Hong W, Huang L, Liu M, Liu H, Zeng H, Deng D, Xin H, Song J (2014). Construction and analysis of high-density linkage map using high-throughput sequencing data. PLoS One.

[23] Mei H, Liu Y, Du Z, Wu K, Cui C, Jiang X, Zhang H, Zheng Y (2017). High-density genetic map construction and gene mapping of basal branching habit and flowers per leaf axil in *Sesame*. Front Plant Sci.

[24] Chakravarti A, Lasher LK, Reefer JE (1991). A maximum likelihood method for estimating genome length using genetic linkage data. Genetics.

[25] Qi B, Yang Y, Yin Y, Xu M, Li H (2014). De novo sequencing, assembly, and analysis of the *Taxodium* 'Zhongshansa' roots and shoots transcriptome in response to short-term waterlogging. BMC Plant Biol.

[26] Yu C, Xu S, Yin Y (2016). Transcriptome analysis of the *Taxodium* 'Zhongshanshan 405′ roots in response to salinity stress. Plant Physiol Biochem.

[27] Broman KW, Wu H, Sen S, Churchill GA (2003). R/qtl: QTL mapping in experimental crosses. Bioinformatics.

[28] Li H, Ribaut J, Li Z, Wang J (2008). Inclusive composite interval mapping (ICIM) for digenic epistasis of quantitative traits in biparental populations. Theor Appl Genet.

[29] Zhang Z, Shang H, Shi Y, Huang L, Li J, Ge Q, Gong J, Liu A, Chen T, Wang D (2016). Construction of a high-density genetic map by specific locus amplified fragment sequencing (SLAF-seq) and its application to quantitative trait loci (QTL) analysis for boll weight in upland cotton (*Gossypium hirsutum*.). BMC Plant Biol.

[30] Zhang Y, Wang L, Xin H, Li D, Ma C, Ding X, Hong W, Zhang X (2013). Construction of a high-density genetic map for sesame based on large scale marker development by specific length amplified fragment (SLAF) sequencing. BMC Plant Biol.

[31] Cheng Y, Yang Y, Wang Z, Qi B, Yin Y, Li H (2015). Development and characterization of EST-SSR markers in *Taxodium* 'zhongshansa. Plant Mol Biol Report.

[32] Yu Y, Zhang X, Yuan J, Li F, Chen X, Zhao Y, Huang L, Zheng H, Xiang J (2015). Genome survey and high-density genetic map construction provide genomic and genetic resources for the Pacific white shrimp *Litopenaeus vannamei*. Sci Rep.

[33] Kovach A, Wegrzyn JL, Parra G, Holt C, Bruening GE, Loopstra CA, Hartigan J, Yandell M, Langley CH, Korf I (2010). The *Pinus taeda* genome is characterized by diverse and highly diverged repetitive sequences. BMC Genomics.

[34] Kusumi J, Tsumura Y, Yoshimaru H, Tachida H (2000). Phylogenetic relationships in Taxodiaceae and Cupressaceae sensu stricto based on matK gene, chlL gene, trnL-trnF IGS region, and trnL intron sequences. Am J Bot.

[35] Moriguchi Y, Ujino-Ihara T, Uchiyama K, Futamura N, Saito M, Ueno S, Matsumoto A, Tani N, Taira H, Shinohara K (2012). The construction of a high-density linkage map for identifying SNP markers that are tightly linked to a nuclear-recessive major gene for male sterility in *Cryptomeria japonica* D. Don. BMC Genomics.

[36] Zhao ZG, Jiang L, Zhang WW, Yu CY, Zhu SS, Xie K, Tian H, Liu LL, Ikehashi H, Wan JM (2007). Fine mapping of *S31*, a gene responsible for hybrid embryo-sac abortion in rice (*Oryza sativa* L.). Planta.

[37] Fishman L, Aagaard J, Tuthill JC (2008). Toward the evolutionary genomics of gametophytic divergence: patterns of transmission ratio distortion in monkeyflower (*Mimulus*) hybrids reveal a complex genetic basis for conspecific pollen precedence. Evolution.

[38] Dai B, Guo H, Huang C, Ahmed MM, Lin Z (2017). Identification and characterization of segregation distortion loci on cotton chromosome 18. Front Plant Sci.

[39] Bradshaw HD, Stettler RF (1994). Molecular genetics of growth and development in Populus. II. Segregation distortion due to genetic load. Theor Appl Genet.

[40] Zhou W, Tang Z, Hou J, Hu N, Yin T (2015). Genetic map construction and detection of genetic loci underlying segregation distortion in an intraspecific cross of *Populus deltoides*. PLoS One.

[41] Kuang H, Richardson TE, Carson SD, Bongarten BC (1998). An allele responsible for seedling death in *Pinus radiata* D. Don. Theor Appl Genet.

[42] Remington DL, O'Malley DM (2000). Whole-genome characterization of embryonic stage inbreeding depression in a selfed loblolly pine family. Genetics.

[43] Cervera MT, Storme V, Ivens B, Gusmao J, Liu BH, Hostyn V, Van Slycken J, Van Montagu M, Boerjan W (2001). Dense genetic linkage maps of three *Populus* species (*Populus deltoides*, *P. nigra* and *P. trichocarpa*) based on AFLP and microsatellite markers. Genetics.

[44] Stirling B, Newcombe G, Vrebalov J, Bosdet I, Bradshaw HD (2001). Suppressed recombination around the *MXC3* locus, a major gene for resistance to poplar leaf rust. Theor Appl Genet.

[45] Marone D, Russo M, Laidò G, De Leonardis A, Mastrangelo A (2013). Plant *nucleotide binding site* -*leucine* -*rich repeat* (*NBS*-*LRR*) genes: active guardians in host defense responses. Int J Mol Sci.

[46] Martin T, Rönnberg-Wästljung A, Stenlid J, Samils B (2016). Identification of a differentially expressed *TIR*-*NBS*-*LRR* gene in a major QTL associated to leaf rust resistance in *Salix*. PLoS One.

[47] Schrader J. (2004). A High-Resolution Transcript Profile across the Wood-Forming Meristem of Poplar Identifies Potential Regulators of Cambial Stem Cell Identity. THE PLANT CELL ONLINE.

[48] Groover AT (2005). What genes make a tree a tree?. Trends Plant Sci.

[49] Groover A, Robischon M (2006). Developmental mechanisms regulating secondary growth in woody plants. Curr Opin Plant Biol.

[50] Li S, Wang J, Zhang L (2015). Inclusive composite interval mapping of QTL by environment interactions in biparental populations. PLoS One.

[51] Remington DL, O'Malley DM (2000). Evaluation of major genetic loci contributing to inbreeding depression for survival and early growth in a selfed family of *Pinus taeda*. Evolution.

[52] Price AH (2006). Believe it or not, QTLs are accurate. Trends Plant Sci.

[53] Shan T, Pang S, Li J, Li X, Su L (2015). construction of a high-density genetic map and mapping of a sex-linked locus for the brown alga *Undaria pinnatifida* (Phaeophyceae) based on large scale marker development by specific length amplified fragment (SLAF) sequencing. BMC Genomics.

[54] Walker JC (1994). Structure and function of the receptor-like protein kinases of higher plants. Plant Mol Biol.

[55] Kucukoglu M, Zheng B. CLE/RLK regulated vascular signalling pathways in plants. Plant Physiol. 2009.

[56] Bryan AC, Obaidi A, Wierzba M, Tax FE (2012). XYLEM INTERMIXED WITH PHLOEM1, a leucine-rich repeat receptor-like kinase required for stem growth and vascular development in *Arabidopsis thaliana*. Planta.

[57] Ávila C, Pérez-Rodríguez J, Cánovas FM (2006). Molecular characterization of a *receptor*-*like protein kinase* gene from pine (*Pinus sylvestris* L.). Planta.

[58] Xu Y (1994). Factors influencing the power of QTL mapping: population size. Journal of Zhejiang Agricultural University.

[59] LI Hui-Hui, ZHANG Lu-Yan, WANG Jian-Kang (2010). Analysis and Answers to Frequently Asked Questions in Quantitative Trait Locus Mapping. ACTA AGRONOMICA SINICA.

