# Supplementary figures and images for: High-density genetic map construction and quantitative trait loci identification for growth traits in (Taxodium distichum var. distichum × T. mucronatum) × T. mucronatum
Source: BMC Plant Biol. 2018 Nov 1;18:263. doi: 10.1186/s12870-018-1493-0 (PMC6474422; doi:10.1186/s12870-018-1493-0)

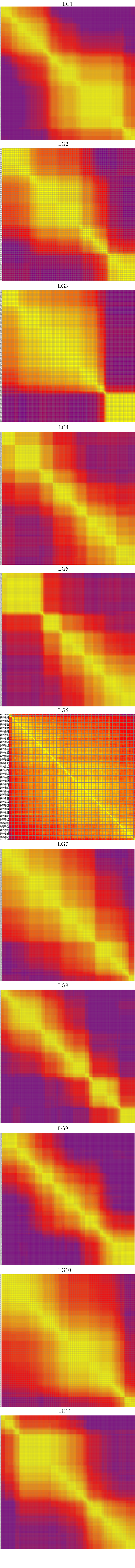

Supplement: Supplementary file 1 — Heat maps of the genetic map. Tif. Each cell represents the recombination rate of two markers. Yellow indicates a lower recombination rate and purple a higher one. (TIF 15423 kb) [file 12870_2018_1493_MOESM2_ESM.tif]
