# Supplementary material for: High-density genetic map construction and quantitative trait loci identification for growth traits in (Taxodium distichum var. distichum × T. mucronatum) × T. mucronatum
Source: BMC Plant Biol. 2018 Nov 1;18:263. doi: 10.1186/s12870-018-1493-0 (PMC6474422; doi:10.1186/s12870-018-1493-0)
Supplement: Supplementary file 3 — Result of the blast analysis of the markers within the SDRs against the unigene database of ‘Zhongshanshan’. (PDF 186 kb) [file 12870_2018_1493_MOESM4_ESM.pdf]

```
BLASTN 2.2.10 [Oct-19-2004]


Reference: Altschul, Stephen F., Thomas L. Madden, Alejandro A. Schaffer,
Jinghui Zhang, Zheng Zhang, Webb Miller, and David J. Lipman (1997),
"Gapped BLAST and PSI-BLAST: a new generation of protein database search
programs",  Nucleic Acids Res. 25:3389-3402.

Query= Marker61462
         (200 letters)

Database: Zhongshanshan 405.Unigene.fasta
           70,312 sequences; 67,915,621 total letters



                                                                 Score    E
Sequences producing significant alignments:                      (bits) Value

CL590Contig1                                                          168   2e-
041

>CL590Contig1
          Length = 3148

 Score =  168 bits (85), Expect = 2e-041
 Identities = 91/93 (97%)
 Strand = Plus / Plus


Query: 4    tgcattgacttctttgaaacaaatgtttgtgagtggagaatggcttaactcatcttattc 63
            |||||||||||||||||||||||||||||||||||||||||||||||||||| |||||||
Sbjct: 1424 tgcattgacttctttgaaacaaatgtttgtgagtggagaatggcttaactcaccttattc 1483


Query: 64   aaagaagcctgatggagaggctgtggcattcat 96
            |||||| ||||||||||||||||||||||||||
Sbjct: 1484 aaagaaacctgatggagaggctgtggcattcat 1516



 Score =  139 bits (70), Expect = 1e-032
 Identities = 88/94 (93%)
 Strand = Plus / Plus


Query: 101  aaaaattactacaaggaggataggctcaaatttgatcccatttgggaaattgttgatagg 160
            ||||| || ||||||| |||||||||||||||||||||||||||||||||||||||||||
Sbjct: 1662 aaaaactattacaagggggataggctcaaatttgatcccatttgggaaattgttgatagg 1721


Query: 161  aggcgaaacaatcaactctaccaacccattcatg 194
            ||| | |||||||||||| |||||||||||||||
Sbjct: 1722 aggtggaacaatcaactccaccaacccattcatg 1755
```

```
BLASTN 2.2.10 [Oct-19-2004]


Reference: Altschul, Stephen F., Thomas L. Madden, Alejandro A. Schaffer,
Jinghui Zhang, Zheng Zhang, Webb Miller, and David J. Lipman (1997),
"Gapped BLAST and PSI-BLAST: a new generation of protein database search
programs",  Nucleic Acids Res. 25:3389-3402.

Query= Marker19772
         (200 letters)

Database: Zhongshanshan 406.Unigene.fasta
           117,776 sequences; 77,714,018 total letters



                                                                 Score    E
Sequences producing significant alignments:                      (bits) Value

comp120213_c1                                                         198   2e-
050


>comp120213_c1
          Length = 1358

 Score =  198 bits (100), Expect = 2e-050
 Identities = 100/100 (100%)
 Strand = Plus / Minus


Query: 1   ttgtatagaccaccagattctttgtgggtcgacggaaatgaagcagctttttagggatac 60
           ||||||||||||||||||||||||||||||||||||||||||||||||||||||||||||
Sbjct: 452 ttgtatagaccaccagattctttgtgggtcgacggaaatgaagcagctttttagggatac 393


Query: 61  gacttcgatcctggtcgacagatgggtttgtgttgtcttg 100
           ||||||||||||||||||||||||||||||||||||||||
Sbjct: 392 gacttcgatcctggtcgacagatgggtttgtgttgtcttg 353



 Score =  192 bits (97), Expect = 1e-048
 Identities = 100/101 (99%)
 Strand = Plus / Minus


Query: 100 gaagcgagcattgctgccattcacaattctgaacctgtattttcttctcttcaccttcat 159
           ||||||||||||||||||||||||||||||||||||||||||||||||||||||||||||
Sbjct: 112 gaagcgagcattgctgccattcacaattctgaacctgtattttcttctcttcaccttcat 53


Query: 160 gtaaggccaggcctttccgttcactatgatggcatctccga 200
           ||||||||||||||||||||||||||| |||||||||||||
Sbjct: 52  gtaaggccaggcctttccgttcactataatggcatctccga 12
```

```
BLASTN 2.2.10 [Oct-19-2004]


Reference: Altschul, Stephen F., Thomas L. Madden, Alejandro A. Schaffer,
Jinghui Zhang, Zheng Zhang, Webb Miller, and David J. Lipman (1997),
"Gapped BLAST and PSI-BLAST: a new generation of protein database search
programs",  Nucleic Acids Res. 25:3389-3402.

Query= Marker68520
         (200 letters)

Database: Zhongshanshan 406.Unigene.fasta
           117,776 sequences; 77,714,018 total letters



                                                                 Score    E
Sequences producing significant alignments:                      (bits) Value

comp105673_c0                                                         198   2e-
050


>comp105673_c0
          Length = 2045

 Score =  198 bits (100), Expect = 2e-050
 Identities = 100/100 (100%)
 Strand = Plus / Plus


Query: 1   aatatatgtgtctgtatcatcattgctgaagcagtttattatggtttgtagtaataggtg 60
           ||||||||||||||||||||||||||||||||||||||||||||||||||||||||||||
Sbjct: 727 aatatatgtgtctgtatcatcattgctgaagcagtttattatggtttgtagtaataggtg 786


Query: 61  tcttacatgtttgtctgtcctctgacaggtgaacgagtat 100
           ||||||||||||||||||||||||||||||||||||||||
Sbjct: 787 tcttacatgtttgtctgtcctctgacaggtgaacgagtat 826



 Score =  182 bits (92), Expect = 1e-045
 Identities = 98/100 (98%)
 Strand = Plus / Plus


Query: 101  ttctgtttagaatttagatctcatgttatgatgtcttgtgaattttgattgcttagtggc 160
            ||||||||||||||||||||||||||||||||||||||||| || |||||||||||||||
Sbjct: 1050 ttctgtttagaatttagatctcatgttatgatgtcttgtgatttatgattgcttagtggc 1109


Query: 161  aattttgggttattttggagtgcgcaattgtttgcatgtg 200
            ||||||||||||||||||||||||||||||||||||||||
Sbjct: 1110 aattttgggttattttggagtgcgcaattgtttgcatgtg 1149
```

```
BLASTN 2.2.10 [Oct-19-2004]


Reference: Altschul, Stephen F., Thomas L. Madden, Alejandro A. Schaffer,
Jinghui Zhang, Zheng Zhang, Webb Miller, and David J. Lipman (1997),
"Gapped BLAST and PSI-BLAST: a new generation of protein database search
programs",  Nucleic Acids Res. 25:3389-3402.

Query= Marker17431
         (200 letters)

Database: Zhongshanshan 406.Unigene.fasta
           117,776 sequences; 77,714,018 total letters



                                                                 Score    E
Sequences producing significant alignments:                      (bits) Value

comp112995_c0                                                         200   5e-
051


>comp112995_c0
          Length = 1056

 Score =  200 bits (101), Expect = 5e-051
 Identities = 101/101 (100%)
 Strand = Plus / Minus


Query: 100 ataatcaggcttggaataatttgccggcaccgttaagtcgcggttattgtcaggcgccca 159
           ||||||||||||||||||||||||||||||||||||||||||||||||||||||||||||
Sbjct: 886 ataatcaggcttggaataatttgccggcaccgttaagtcgcggttattgtcaggcgccca 827


Query: 160 gcagacgaggttcccacttgaaaagctcaccaccattggtt 200
           |||||||||||||||||||||||||||||||||||||||||
Sbjct: 826 gcagacgaggttcccacttgaaaagctcaccaccattggtt 786
```

```
BLASTN 2.2.10 [Oct-19-2004]


Reference: Altschul, Stephen F., Thomas L. Madden, Alejandro A. Schaffer,
Jinghui Zhang, Zheng Zhang, Webb Miller, and David J. Lipman (1997),
"Gapped BLAST and PSI-BLAST: a new generation of protein database search
programs",  Nucleic Acids Res. 25:3389-3402.

Query= Marker32187
         (200 letters)

Database: Zhongshanshan 405.Unigene.fasta
           70,312 sequences; 67,915,621 total letters



                                                                 Score    E
Sequences producing significant alignments:                      (bits) Value

T2_Unigene_BMK.15009                                                  182   1e-
045


>T2_Unigene_BMK.15009
          Length = 318

 Score =  182 bits (92), Expect = 1e-045
 Identities = 98/100 (98%)
 Strand = Plus / Plus


Query: 1   tgcattcatttcatctctcaattacaacaaaggaatagaaaccctaataggtagcccatg 60
           ||||||||||||||||||||||||||||||||||||||||||||||||||||||||||||
Sbjct: 61  tgcattcatttcatctctcaattacaacaaaggaatagaaaccctaataggtagcccatg 120


Query: 61  gctctctctttcacaaaaagaagtagccaactgtgtgata 100
           |||||||||| |||||||||||||||||||| |||||||||
Sbjct: 121 gctctctcttccacaaaaagaagtagccaattgtgtgata 160
```

```
BLASTN 2.2.10 [Oct-19-2004]


Reference: Altschul, Stephen F., Thomas L. Madden, Alejandro A. Schaffer,
Jinghui Zhang, Zheng Zhang, Webb Miller, and David J. Lipman (1997),
"Gapped BLAST and PSI-BLAST: a new generation of protein database search
programs",  Nucleic Acids Res. 25:3389-3402.
```

Query= Marker66426

```
         (200 letters)
```

Database: **Zhongshanshan 405**.Unigene.fasta

```
           70,312 sequences; 67,915,621 total letters



                                                                 Score    E
Sequences producing significant alignments:                      (bits) Value
```

CL1535Contig1 198 2e-050

```
>CL1535Contig1
          Length = 2741

 Score =  198 bits (100), Expect = 2e-050
 Identities = 100/100 (100%)
 Strand = Plus / Minus


Query: 1    ccccactgtggatggaaaatcgtaactcctgaattcggttgacgtttgtgatgcttcttg 60
            ||||||||||||||||||||||||||||||||||||||||||||||||||||||||||||
Sbjct: 2306 ccccactgtggatggaaaatcgtaactcctgaattcggttgacgtttgtgatgcttcttg 2247


Query: 61   gaaaatgcttgtctgctcaaatgttgcatagtcactgtca 100
            ||||||||||||||||||||||||||||||||||||||||
Sbjct: 2246 gaaaatgcttgtctgctcaaatgttgcatagtcactgtca 2207



 Score =  198 bits (100), Expect = 2e-050
 Identities = 100/100 (100%)
 Strand = Plus / Minus


Query: 101  atcctttctacactcaggggtttcagacctgtcaaaagctcaaccaggacaacaccaaaa 160
            ||||||||||||||||||||||||||||||||||||||||||||||||||||||||||||
Sbjct: 1975 atcctttctacactcaggggtttcagacctgtcaaaagctcaaccaggacaacaccaaaa 1916


Query: 161  ctgaatacatcacttttgtcagtgagctgataagtctgga 200
            ||||||||||||||||||||||||||||||||||||||||
Sbjct: 1915 ctgaatacatcacttttgtcagtgagctgataagtctgga 1876
```

BLASTN 2.2.10 [Oct-19-2004]

Reference: Altschul, Stephen F., Thomas L. Madden, Alejandro A. Schaffer,
Jinghui Zhang, Zheng Zhang, Webb Miller, and David J. Lipman (1997),
"Gapped BLAST and PSI-BLAST: a new generation of protein database search
programs", Nucleic Acids Res. 25:3389-3402.

```
Query= Marker70472
         (200 letters)

Database: Zhongshanshan 406.Unigene.fasta

           117,776 sequences; 77,714,018 total letters



                                                                  Score    E
Sequences producing significant alignments:                      (bits) Value

comp36128_c0                                                           176   8e-
044


>comp36128_c0
          Length = 269

 Score =  176 bits (89), Expect = 8e-044
 Identities = 95/97 (97%)
 Strand = Plus / Minus


Query: 1  tttctcctcctgggttcttttgttgttgttaacaagcttgaaattttattttatctactg 60
          |||||||||||||||||||||||||||||||||||||||||||||| |||||||||||||
Sbjct: 97 tttctcctcctgggttcttttgttgttgttaacaagcttgaaatttaattttatctactg 38


Query: 61 atgctccacctattatgctgtctgaattcattcatga 97
          |||||||||||||||||||||| ||||||||||||||
Sbjct: 37 atgctccacctattatgctgtccgaattcattcatga 1
```

```
BLASTN 2.2.10 [Oct-19-2004]


Reference: Altschul, Stephen F., Thomas L. Madden, Alejandro A. Schaffer,
Jinghui Zhang, Zheng Zhang, Webb Miller, and David J. Lipman (1997),
"Gapped BLAST and PSI-BLAST: a new generation of protein database search
programs",  Nucleic Acids Res. 25:3389-3402.

Query= Marker52261
         (200 letters)

Database: Zhongshanshan 406.Unigene.fasta

           117,776 sequences; 77,714,018 total letters



                                                                 Score    E
Sequences producing significant alignments:                      (bits) Value

comp116882_c0                                                         182   1e-
045


>comp116882_c0
          Length = 1992

 Score =  182 bits (92), Expect = 1e-045
 Identities = 92/92 (100%)
 Strand = Plus / Minus


Query: 109 caggtcagaggtttataaagattgccaaatctcctgtgtctcccagtttagcatttccat 168
           ||||||||||||||||||||||||||||||||||||||||||||||||||||||||||||
Sbjct: 870 caggtcagaggtttataaagattgccaaatctcctgtgtctcccagtttagcatttccat 811


Query: 169 ttatgattactgccttgggcatagaactcgta 200
           ||||||||||||||||||||||||||||||||
Sbjct: 810 ttatgattactgccttgggcatagaactcgta 779
```

```
BLASTN 2.2.10 [Oct-19-2004]


Reference: Altschul, Stephen F., Thomas L. Madden, Alejandro A. Schaffer,
Jinghui Zhang, Zheng Zhang, Webb Miller, and David J. Lipman (1997),
"Gapped BLAST and PSI-BLAST: a new generation of protein database search
programs",  Nucleic Acids Res. 25:3389-3402.

Query= Marker87936
         (200 letters)

Database: Zhongshanshan 406.Unigene.fasta
           117,776 sequences; 77,714,018 total letters



                                                                 Score    E
Sequences producing significant alignments:                      (bits) Value

comp103289_c0                                                        190   5e-
048


>comp103289_c0
          Length = 1145

 Score =  190 bits (96), Expect = 5e-048
 Identities = 99/100 (99%)
 Strand = Plus / Plus


Query: 1   taaagagacattttgtccaagcccacagacatgaatgagagcatttaattggttcagtgt 60
           |||||||||||||||||| ||||||||||||||||||||||||||||||||||||||||||
Sbjct: 733 taaagagacattttgtccgagcccacagacatgaatgagagcatttaattggttcagtgt 792


Query: 61  tgtgatatttgtgcactcttctgctaaagaagcgtaagta 100
           ||||||||||||||||||||||||||||||||||||||||
Sbjct: 793 tgtgatatttgtgcactcttctgctaaagaagcgtaagta 832



 Score =  165 bits (83), Expect = 3e-040
 Identities = 83/83 (100%)
 Strand = Plus / Plus


Query: 101  tttttcgtatcagttactgtgttttccctggattacacgttttgatcgaactcattcag 160
            ||||||||||||||||||||||||||||||||||||||||||||||||||||||||||||
Sbjct: 1063 tttttcgtatcagttactgtgttttccctggattacacgttttgatcgaactcattcag 1122


Query: 161  ctatggttttatttgggttttgg 183
            |||||||||||||||||||||||
Sbjct: 1123 ctatggttttatttgggttttgg 1145
```

BLASTN 2.2.10 [Oct-19-2004]

Reference: Altschul, Stephen F., Thomas L. Madden, Alejandro A. Schaffer, Jinghui Zhang, Zheng Zhang, Webb Miller, and David J. Lipman (1997), "Gapped BLAST and PSI-BLAST: a new generation of protein database search programs", Nucleic Acids Res. 25:3389-3402.

Query= **Marker2404**
(200 letters)

Database: **Zhongshanshan 405**.Unigene.fasta
70,312 sequences; 67,915,621 total letters

```
                                                                 Score    E
Sequences producing significant alignments:                      (bits) Value

CL25873Contig1                                                       167   6e-
041

>CL25873Contig1
          Length = 1047

 Score =  167 bits (84), Expect = 6e-041
 Identities = 84/84 (100%)
 Strand = Plus / Plus


Query: 117 ctggaagctcctaagccatctggagttccataataagctgctcgtgaatgtatgaattgg 176
           ||||||||||||||||||||||||||||||||||||||||||||||||||||||||||||
Sbjct: 830 ctggaagctcctaagccatctggagttccataataagctgctcgtgaatgtatgaattgg 889


Query: 177 gaactacagtgaccaatgctgaac 200
           ||||||||||||||||||||||||
Sbjct: 890 gaactacagtgaccaatgctgaac 913
```

```
BLASTN 2.2.10 [Oct-19-2004]


Reference: Altschul, Stephen F., Thomas L. Madden, Alejandro A. Schaffer,
Jinghui Zhang, Zheng Zhang, Webb Miller, and David J. Lipman (1997),
"Gapped BLAST and PSI-BLAST: a new generation of protein database search
programs",  Nucleic Acids Res. 25:3389-3402.

Query= Marker46425
         (200 letters)

Database: Zhongshanshan 405.Unigene.fasta
           70,312 sequences; 67,915,621 total letters



                                                                 Score    E
Sequences producing significant alignments:                      (bits) Value

T3_Unigene_BMK.39289                                                  190   4e-
048


>T3_Unigene_BMK.39289
          Length = 278

 Score =  190 bits (96), Expect = 4e-048
 Identities = 99/100 (99%)
 Strand = Plus / Plus


Query: 1   ttacttggaggtttttatacagtgggaagaataacaatgggtatttataccgtccctgt 60
           ||||||||||||||||||||||||| |||||||||||||||||||||||||||||||||
Sbjct: 96  ttacttggaggtttttatacagtggaaagaataacaatgggtatttataccgtccctgt 155


Query: 61  cggtgacatcacaggacgttattatctgcggaaagaaact 100
           ||||||||||||||||||||||||||||||||||||||||
Sbjct: 156 cggtgacatcacaggacgttattatctgcggaaagaaact 195
```

```
BLASTN 2.2.10 [Oct-19-2004]


Reference: Altschul, Stephen F., Thomas L. Madden, Alejandro A. Schaffer,
Jinghui Zhang, Zheng Zhang, Webb Miller, and David J. Lipman (1997),
"Gapped BLAST and PSI-BLAST: a new generation of protein database search
programs",  Nucleic Acids Res. 25:3389-3402.

Query= Marker36328
         (200 letters)

Database: Zhongshanshan 406.Unigene.fasta
           117,776 sequences; 77,714,018 total letters



                                                                 Score    E
Sequences producing significant alignments:                      (bits) Value

comp111914_c0                                                       198   2e-
050


>comp111914_c0
          Length = 1107

 Score =  198 bits (100), Expect = 2e-050
 Identities = 100/100 (100%)
 Strand = Plus / Minus


Query: 1   cggctgcggtgcagagcgtggcggagctgaagagccgtgaggaatgggaggcctgtgatg 60
           ||||||||||||||||||||||||||||||||||||||||||||||||||||||||||||
Sbjct: 647 cggctgcggtgcagagcgtggcggagctgaagagccgtgaggaatgggaggcctgtgatg 588


Query: 61  tgagcaatccgattcgcctttacaaaggcggccttgattc 100
           ||||||||||||||||||||||||||||||||||||||||
Sbjct: 587 tgagcaatccgattcgcctttacaaaggcggccttgattc 548



 Score =  198 bits (100), Expect = 2e-050
 Identities = 100/100 (100%)
 Strand = Plus / Minus


Query: 101 atgactccatttcacctgacggatttgcttcagtttctgaaacttcagttttacgcactt 160
           ||||||||||||||||||||||||||||||||||||||||||||||||||||||||||||
Sbjct: 310 atgactccatttcacctgacggatttgcttcagtttctgaaacttcagttttacgcactt 251


Query: 161 gactttaagttgacagctatggctgctactgtttggatga 200
           ||||||||||||||||||||||||||||||||||||||||
Sbjct: 250 gactttaagttgacagctatggctgctactgtttggatga 211




BLASTN 2.2.10 [Oct-19-2004]
```

Reference: Altschul, Stephen F., Thomas L. Madden, Alejandro A. Schaffer, Jinghui Zhang, Zheng Zhang, Webb Miller, and David J. Lipman (1997), "Gapped BLAST and PSI-BLAST: a new generation of protein database search programs", Nucleic Acids Res. 25:3389-3402.

Query= **Marker25953**
(200 letters)

Database: **Zhongshanshan 406**.Unigene.fasta
117,776 sequences; 77,714,018 total letters

```
                                                                 Score    E
Sequences producing significant alignments:                      (bits) Value

comp81440_c1                                                          190   5e-
048
```

>comp81440_c1
Length = 242

Score = 190 bits (96), Expect = 5e-048
Identities = 99/100 (99%)
Strand = Plus / Plus

```
Query: 96  agaaaatgaaagatatggtatggagacataatgcaacaatggaaacagagaggaagagac 155
           ||||||||||||||||||||||||||||||||||||||||||||||||||||||||||||
Sbjct: 143 agaaaatgaaagatatggtatggagacataatgcaacaatggaaacagagaggaagagac 202


Query: 156 tccccttcaacaccaagagaacaagagcctgccagcgtaa 195
           |||||||||||||||||||||||||||||||||| ||||
Sbjct: 203 tccccttcaacaccaagagaacaagagcctgccagggtaa 242
```

```
BLASTN 2.2.10 [Oct-19-2004]


Reference: Altschul, Stephen F., Thomas L. Madden, Alejandro A. Schaffer,
Jinghui Zhang, Zheng Zhang, Webb Miller, and David J. Lipman (1997),
"Gapped BLAST and PSI-BLAST: a new generation of protein database search
programs",  Nucleic Acids Res. 25:3389-3402.

Query= Marker40499
         (200 letters)

Database: Zhongshanshan 406.Unigene.fasta
           117,776 sequences; 77,714,018 total letters



                                                                 Score    E
Sequences producing significant alignments:                      (bits) Value

comp127474_c0                                                         176   8e-
044

>comp127474_c0
          Length = 6084

 Score =  176 bits (89), Expect = 8e-044
 Identities = 99/101 (98%), Gaps = 1/101 (0%)
 Strand = Plus / Minus


Query: 101  gcaaaaaaatgcctcctggtcagaattgcaaaactaaaaagtgtattgttgttt-aaaaa 159
            |||||||||||||||||||||||||||||||||||||||||||||||||||||| |||||
Sbjct: 3839 gcaaaaaaatgcctcctggtcagaattgcaaaactaaaaagtgtattgttgtttaaaaaa 3780


Query: 160  aagtgatgccccgacagacctgtatttgattatgaaatcca 200
            ||||||||||||| |||||||||||||||||||||||||||
Sbjct: 3779 aagtgatgccccggcagacctgtatttgattatgaaatcca 3739



 Score =  159 bits (80), Expect = 2e-038
 Identities = 95/100 (95%)
 Strand = Plus / Minus


Query: 1    caaatcgagcagcatctatccctctaacagacattcctctctggatgagaaaacgaacag 60
            |||||||||||||||||||||||||||||||| ||||||| ||| ||||||||||||| ||
Sbjct: 4168 caaatcgagcagcatctatccctctaacagaaattcctccctgaatgagaaaacgaagag 4109


Query: 61   cataaattcaatgtaatgtgaggatcagcaggacaaatac 100
            |||||||||||||||||||||||||||||||| ||||||||
Sbjct: 4108 cataaattcaatgtaatgtgaggatcagcagcacaaatac 4069
```

```
BLASTN 2.2.10 [Oct-19-2004]


Reference: Altschul, Stephen F., Thomas L. Madden, Alejandro A. Schaffer,
Jinghui Zhang, Zheng Zhang, Webb Miller, and David J. Lipman (1997),
"Gapped BLAST and PSI-BLAST: a new generation of protein database search
programs",  Nucleic Acids Res. 25:3389-3402.

Query= Marker33615
         (200 letters)

Database: Zhongshanshan 406.Unigene.fasta
           117,776 sequences; 77,714,018 total letters



                                                                 Score    E
Sequences producing significant alignments:                      (bits) Value

comp122759_c1                                                         198   2e-
050


>comp122759_c1
          Length = 548

 Score =  198 bits (100), Expect = 2e-050
 Identities = 100/100 (100%)
 Strand = Plus / Plus


Query: 101 ttcgtcgacaagaatatgaaatgatgcctaggagatttgagtgggctgaggattcaatat 160
           ||||||||||||||||||||||||||||||||||||||||||||||||||||||||||||
Sbjct: 69  ttcgtcgacaagaatatgaaatgatgcctaggagatttgagtgggctgaggattcaatat 128


Query: 161 ataaccaggatgcaattgagctctctggtggaagataaga 200
           ||||||||||||||||||||||||||||||||||||||||
Sbjct: 129 ataaccaggatgcaattgagctctctggtggaagataaga 168
```

```
BLASTN 2.2.10 [Oct-19-2004]


Reference: Altschul, Stephen F., Thomas L. Madden, Alejandro A. Schaffer,
Jinghui Zhang, Zheng Zhang, Webb Miller, and David J. Lipman (1997),
"Gapped BLAST and PSI-BLAST: a new generation of protein database search
programs",  Nucleic Acids Res. 25:3389-3402.

Query= Marker54711
         (200 letters)

Database: Zhongshanshan 406.Unigene.fasta
           117,776 sequences; 77,714,018 total letters



                                                                 Score    E
Sequences producing significant alignments:                      (bits) Value

comp107777_c0                                                         200   5e-
051


>comp107777_c0
          Length = 2939

 Score =  200 bits (101), Expect = 5e-051
 Identities = 101/101 (100%)
 Strand = Plus / Plus


Query: 100 atcccataatgggcctaggtttgttgctcttgggtaagagtttcttgctggaccatgtta 159
           ||||||||||||||||||||||||||||||||||||||||||||||||||||||||||||
Sbjct: 825 atcccataatgggcctaggtttgttgctcttgggtaagagtttcttgctggaccatgtta 884


Query: 160 tcttgtcagatttggcactataaggcttttgttcagtttct 200
           |||||||||||||||||||||||||||||||||||||||||
Sbjct: 885 tcttgtcagatttggcactataaggcttttgttcagtttct 925



 Score =  129 bits (65), Expect = 2e-029
 Identities = 65/65 (100%)
 Strand = Plus / Plus


Query: 12  atgatcctagtaaagatccaaatatgtcaggtgatccatacaagacactgtttgttgcaa 71
           ||||||||||||||||||||||||||||||||||||||||||||||||||||||||||||
Sbjct: 680 atgatcctagtaaagatccaaatatgtcaggtgatccatacaagacactgtttgttgcaa 739


Query: 72  gactt 76
           |||||
Sbjct: 740 gactt 744
```

```
BLASTN 2.2.10 [Oct-19-2004]


Reference: Altschul, Stephen F., Thomas L. Madden, Alejandro A. Schaffer,
Jinghui Zhang, Zheng Zhang, Webb Miller, and David J. Lipman (1997),
"Gapped BLAST and PSI-BLAST: a new generation of protein database search
programs",  Nucleic Acids Res. 25:3389-3402.

Query= Marker1862
         (200 letters)

Database: Zhongshanshan 406.Unigene.fasta
           117,776 sequences; 77,714,018 total letters



                                                                 Score    E
Sequences producing significant alignments:                      (bits) Value

comp113075_c0                                                         198   2e-
050


>comp113075_c0
          Length = 1124

 Score =  198 bits (100), Expect = 2e-050
 Identities = 100/100 (100%)
 Strand = Plus / Minus


Query: 1   cctcttccccttctacctccaaagacaaaagcttttcttcgtcttcatctaggaaggaac 60
           ||||||||||||||||||||||||||||||||||||||||||||||||||||||||||||
Sbjct: 769 cctcttccccttctacctccaaagacaaaagcttttcttcgtcttcatctaggaaggaac 710


Query: 61  tgcccacttcaaaagcatcctccgccattgtcaagaccaa 100
           ||||||||||||||||||||||||||||||||||||||||
Sbjct: 709 tgcccacttcaaaagcatcctccgccattgtcaagaccaa 670



 Score =  198 bits (100), Expect = 2e-050
 Identities = 100/100 (100%)
 Strand = Plus / Minus


Query: 101 gggagccattctcagaagaaaatgttgaccgactctaagttcactcgccctttgtctgtg 160
           ||||||||||||||||||||||||||||||||||||||||||||||||||||||||||||
Sbjct: 440 gggagccattctcagaagaaaatgttgaccgactctaagttcactcgccctttgtctgtg 381


Query: 161 gtgcttaagagtgagcaggaacttttgcagcagaataaag 200
           ||||||||||||||||||||||||||||||||||||||||
Sbjct: 380 gtgcttaagagtgagcaggaacttttgcagcagaataaag 341
```
