# Supplementary material for: High-density genetic map construction and quantitative trait loci identification for growth traits in (Taxodium distichum var. distichum × T. mucronatum) × T. mucronatum
Source: BMC Plant Biol. 2018 Nov 1;18:263. doi: 10.1186/s12870-018-1493-0 (PMC6474422; doi:10.1186/s12870-018-1493-0)
Supplement: Supplementary file 5 — The characters of 7 consensus loci associated with growth-related traits across years detected by the CIM method. (DOC 215 kb) [file 12870_2018_1493_MOESM6_ESM.doc]

The characters of 7 consensus loci associated with growth-related traits across years detected by the CIM method.

| Name | Trait | LG | Position(cM) | Locus | LOD | ADD | PVE(%) |
| --- | --- | --- | --- | --- | --- | --- | --- |
| *q2-1* | SH2016 | 2 | 4.622 | Marker22206 | 2.76 | 54.17 | 6.99 |
|  |  | 2 | 4.622 | Marker54415 | 2.76 | 54.17 | 6.99 |
|  | SH2015 | 2 | 0 | Marker29034 | 3.03 | 33.47 | 5.67 |
|  |  | 2 | 0 | Marker32141 | 3.03 | 33.47 | 5.67 |
|  |  | 2 | 0 | Marker13210 | 3.03 | 33.47 | 5.67 |
|  |  | 2 | 0 | Marker29918 | 3.03 | 33.47 | 5.67 |
|  |  | 2 | 0 | Marker12102 | 3.03 | 33.47 | 5.67 |
|  |  | 2 | 0 | Marker69386 | 3.03 | 33.47 | 5.67 |
|  |  | 2 | 0 | Marker68984 | 3.03 | 33.47 | 5.67 |
|  |  | 2 | 0 | Marker14555 | 3.03 | 33.47 | 5.67 |
|  |  | 2 | 0 | Marker75976 | 3.03 | 33.47 | 5.67 |
|  |  | 2 | 0 | Marker75396 | 3.03 | 33.47 | 5.67 |
|  |  | 2 | 0 | Marker7427 | 3.03 | 33.47 | 5.67 |
|  |  | 2 | 0 | Marker46110 | 3.03 | 33.47 | 5.67 |
|  |  | 2 | 0 | Marker51215 | 3.03 | 33.47 | 5.67 |
|  |  | 2 | 0 | Marker7275 | 3.03 | 33.47 | 5.67 |
|  |  | 2 | 0 | Marker18813 | 3.03 | 33.47 | 5.67 |
|  |  | 2 | 0 | Marker85948 | 3.03 | 33.47 | 5.67 |
|  |  | 2 | 0 | Marker7528 | 3.03 | 33.47 | 5.67 |
|  |  | 2 | 0 | Marker11449 | 3.03 | 33.47 | 5.67 |
|  |  | 2 | 0 | Marker46222 | 3.03 | 33.47 | 5.67 |
|  |  | 2 | 0 | Marker95144 | 3.03 | 33.47 | 5.67 |
|  |  | 2 | 0 | Marker6082 | 3.03 | 33.47 | 5.67 |
|  | SH2014 | 2 | 3.956 | Marker137348 | 2.14 | 16.90 | 4.52 |
|  |  | 2 | 3.956 | Marker37943 | 2.14 | 16.90 | 4.52 |
|  |  | 2 | 3.956 | Marker28548 | 2.14 | 16.90 | 4.52 |
|  |  | 2 | 3.956 | Marker36758 | 2.14 | 16.90 | 4.52 |
|  |  | 2 | 3.956 | Marker110085 | 2.14 | 16.90 | 4.52 |
|  |  | 2 | 3.956 | Marker29387 | 2.14 | 16.90 | 4.52 |
|  |  | 2 | 3.956 | Marker18610 | 2.14 | 16.90 | 4.52 |
|  |  | 2 | 3.956 | Marker4629 | 2.14 | 16.90 | 4.52 |
|  |  | 2 | 3.956 | Marker15254 | 2.14 | 16.90 | 4.52 |
|  |  | 2 | 3.956 | Marker49348 | 2.14 | 16.90 | 4.52 |
|  | BD2015 | 2 | 0 | Marker29034 | 2.66 | 7.87 | 5.21 |
|  |  | 2 | 0 | Marker32141 | 2.66 | 7.87 | 5.21 |
|  |  | 2 | 0 | Marker13210 | 2.66 | 7.87 | 5.21 |
|  |  | 2 | 0 | Marker29918 | 2.66 | 7.87 | 5.21 |
|  |  | 2 | 0 | Marker12102 | 2.66 | 7.87 | 5.21 |
|  |  | 2 | 0 | Marker69386 | 2.66 | 7.87 | 5.21 |
|  |  | 2 | 0 | Marker68984 | 2.66 | 7.87 | 5.21 |
|  |  | 2 | 0 | Marker14555 | 2.66 | 7.87 | 5.21 |
|  |  | 2 | 0 | Marker75976 | 2.66 | 7.87 | 5.21 |
|  |  | 2 | 0 | Marker75396 | 2.66 | 7.87 | 5.21 |
|  |  | 2 | 0 | Marker7427 | 2.66 | 7.87 | 5.21 |
|  |  | 2 | 0 | Marker46110 | 2.66 | 7.87 | 5.21 |
|  |  | 2 | 0 | Marker51215 | 2.66 | 7.87 | 5.21 |
|  |  | 2 | 0 | Marker7275 | 2.66 | 7.87 | 5.21 |
|  |  | 2 | 0 | Marker18813 | 2.66 | 7.87 | 5.21 |
|  |  | 2 | 0 | Marker85948 | 2.66 | 7.87 | 5.21 |
|  |  | 2 | 0 | Marker7528 | 2.66 | 7.87 | 5.21 |
|  |  | 2 | 0 | Marker11449 | 2.66 | 7.87 | 5.21 |
|  |  | 2 | 0 | Marker46222 | 2.66 | 7.87 | 5.21 |
|  |  | 2 | 0 | Marker95144 | 2.66 | 7.87 | 5.21 |
|  |  | 2 | 0 | Marker6082 | 2.66 | 7.87 | 5.21 |
|  | BD2014 | 2 | 0 | Marker6082 | 2.30 | 4.54 | 4.55 |
|  |  | 2 | 0 | Marker95144 | 2.30 | 4.54 | 4.55 |
|  |  | 2 | 0 | Marker46222 | 2.30 | 4.54 | 4.55 |
|  |  | 2 | 0 | Marker11449 | 2.30 | 4.54 | 4.55 |
|  |  | 2 | 0 | Marker7528 | 2.30 | 4.54 | 4.55 |
|  |  | 2 | 0 | Marker85948 | 2.30 | 4.54 | 4.55 |
|  |  | 2 | 0 | Marker18813 | 2.30 | 4.54 | 4.55 |
|  |  | 2 | 0 | Marker7275 | 2.30 | 4.54 | 4.55 |
|  |  | 2 | 0 | Marker51215 | 2.30 | 4.54 | 4.55 |
|  |  | 2 | 0 | Marker46110 | 2.30 | 4.54 | 4.55 |
|  |  | 2 | 0 | Marker7427 | 2.30 | 4.54 | 4.55 |
|  |  | 2 | 0 | Marker75396 | 2.30 | 4.54 | 4.55 |
|  |  | 2 | 0 | Marker75976 | 2.30 | 4.54 | 4.55 |
|  |  | 2 | 0 | Marker14555 | 2.30 | 4.54 | 4.55 |
|  |  | 2 | 0 | Marker68984 | 2.30 | 4.54 | 4.55 |
|  |  | 2 | 0 | Marker69386 | 2.30 | 4.54 | 4.55 |
|  |  | 2 | 0 | Marker12102 | 2.30 | 4.54 | 4.55 |
|  |  | 2 | 0 | Marker29918 | 2.30 | 4.54 | 4.55 |
|  |  | 2 | 0 | Marker13210 | 2.30 | 4.54 | 4.55 |
|  |  | 2 | 0 | Marker32141 | 2.30 | 4.54 | 4.55 |
|  |  | 2 | 0 | Marker29034 | 2.30 | 4.54 | 4.55 |
|  | CW2015 | 2 | 0 | Marker6082 | 2.44 | 13.20 | 4.72 |
|  |  | 2 | 0 | Marker46222 | 2.44 | 13.20 | 4.72 |
|  |  | 2 | 0 | Marker46110 | 2.44 | 13.20 | 4.72 |
|  |  | 2 | 0 | Marker14555 | 2.44 | 13.20 | 4.72 |
|  |  | 2 | 0 | Marker18813 | 2.44 | 13.20 | 4.72 |
|  |  | 2 | 0 | Marker85948 | 2.44 | 13.20 | 4.72 |
|  |  | 2 | 0 | Marker7528 | 2.44 | 13.20 | 4.72 |
|  |  | 2 | 0 | Marker11449 | 2.44 | 13.20 | 4.72 |
|  |  | 2 | 0 | Marker29034 | 2.44 | 13.20 | 4.72 |
|  |  | 2 | 0 | Marker7427 | 2.44 | 13.20 | 4.72 |
|  |  | 2 | 0 | Marker75976 | 2.44 | 13.20 | 4.72 |
|  |  | 2 | 0 | Marker95144 | 2.44 | 13.20 | 4.72 |
|  |  | 2 | 0 | Marker51215 | 2.44 | 13.20 | 4.72 |
|  |  | 2 | 0 | Marker32141 | 2.44 | 13.20 | 4.72 |
|  |  | 2 | 0 | Marker68984 | 2.44 | 13.20 | 4.72 |
|  |  | 2 | 0 | Marker12102 | 2.44 | 13.20 | 4.72 |
|  |  | 2 | 0 | Marker29918 | 2.44 | 13.20 | 4.72 |
|  |  | 2 | 0 | Marker69386 | 2.44 | 13.20 | 4.72 |
|  |  | 2 | 0 | Marker75396 | 2.44 | 13.20 | 4.72 |
|  |  | 2 | 0 | Marker13210 | 2.44 | 13.20 | 4.72 |
|  |  | 2 | 0 | Marker7275 | 2.44 | 13.20 | 4.72 |
| *q2-2* | DBH2016 | 2 | 16.248 | Marker15435 | 2.57 | 1.12 | 7.81 |
|  | CW2014 | 2 | 20.954 | Marker61488 | 2.96 | 821.08 | 3.93 |
|  |  | 2 | 19.622 | Marker50666 | 2.19 | 558.37 | 1.82 |
| *q2-3* | DBH2015 | 2 | 39.58 | Marker67259 | 3.00 | 0.67 | 7.54 |
|  |  | 2 | 39.58 | Marker116898 | 3.00 | 0.67 | 7.54 |
| *q4-1* | SH2016 | 4 | 83.936 | Marker16050 | 2.99 | 61.98 | 9.15 |
|  |  | 4 | 83.937 | Marker6401 | 2.99 | 61.98 | 9.15 |
|  |  | 4 | 83.937 | Marker20492 | 2.99 | 61.98 | 9.15 |
|  | SH2015 | 4 | 83.936 | Marker16050 | 3.96 | 43.71 | 9.67 |
|  |  | 4 | 83.937 | Marker6401 | 3.96 | 43.71 | 9.67 |
|  |  | 4 | 83.937 | Marker20492 | 3.96 | 43.71 | 9.67 |
|  | SH2014 | 4 | 83.936 | Marker16050 | 3.53 | 23.46 | 8.71 |
|  |  | 4 | 83.937 | Marker6401 | 3.53 | 23.46 | 8.71 |
|  |  | 4 | 83.937 | Marker20492 | 3.53 | 23.46 | 8.71 |
|  | BD2015 | 4 | 83.936 | Marker16050 | 2.80 | 9.22 | 7.15 |
|  |  | 4 | 83.937 | Marker6401 | 2.80 | 9.22 | 7.15 |
|  |  | 4 | 83.937 | Marker20492 | 2.80 | 9.22 | 7.15 |
|  | BD2014 | 4 | 83.937 | Marker20492 | 2.83 | 5.77 | 7.35 |
|  |  | 4 | 83.937 | Marker6401 | 2.83 | 5.77 | 7.35 |
|  |  | 4 | 83.936 | Marker16050 | 2.83 | 5.77 | 7.35 |
| *q6-1* | SH2016 | 6 | 48.413 | Marker18748 | 3.73 | 71.93 | 12.32 |
|  |  | 6 | 48.425 | Marker130041 | 3.31 | 70.29 | 11.76 |
| *q6-2* | SH2015 | 6 | 60.837 | Marker54767 | 5.85 | 50.85 | 13.09 |
|  | SH2014 | 6 | 60.837 | Marker54767 | 3.98 | 25.30 | 10.13 |
|  | BD2015 | 6 | 60.837 | Marker54767 | 5.48 | 12.33 | 12.78 |
|  | BD2014 | 6 | 60.837 | Marker54767 | 4.82 | 7.19 | 11.42 |
|  | DBH2016 | 6 | 60.837 | Marker54767 | 5.09 | 1.31 | 10.76 |
|  | DBH2015 | 6 | 58.635 | Marker44609 | 3.53 | 0.79 | 10.45 |
|  |  | 6 | 58.897 | Marker207336 | 3.29 | 0.74 | 9.29 |
|  | CW2015 | 6 | 60.837 | Marker54767 | 6.00 | 3490.11 | 13.35 |
|  | CW2014 | 6 | 60.837 | Marker54767 | 3.45 | 1321.97 | 10.20 |
| *q8-1* | DBH2015 | 8 | 41.861 | Marker33252 | 2.78 | 0.66 | 7.29 |
|  |  | 8 | 41.861 | Marker43204 | 2.78 | 0.66 | 7.29 |

‘Name’ indicates the name of the QTL defined by the chromosome number. Trait: The traits-years of QTL is a composite of the influenced trait: seedling height(SH), basal diameter(BD) , crown width(CW) and the diameter at breast height (DBH) followed by the years. ‘LG’ indicates chromosome. ‘Position’ indicates the position of SLAF marker on chromosome in centimorgans. ‘Locus’ indicates the name of the SALF marker. ‘LOD’ indicates the average logarithm of odds score. ‘Add’ indicates the additive effect. ‘PVE’ indicates the average phenotypic variance explained by related QTL.
