# Supplementary material for: High-density genetic map construction and quantitative trait loci identification for growth traits in (Taxodium distichum var. distichum × T. mucronatum) × T. mucronatum
Source: BMC Plant Biol. 2018 Nov 1;18:263. doi: 10.1186/s12870-018-1493-0 (PMC6474422; doi:10.1186/s12870-018-1493-0)
Supplement: Supplementary file 6 — The characters of 13 consensus loci associated with growth-related traits across various years detected by the ICIM method. (DOC 60 kb) [file 12870_2018_1493_MOESM7_ESM.doc]

***The characters of 13 consensus loci associated with growth-related traits across various years detected by the ICIM method***

| Name | Trait | LG | Position (cM) | Marker interval | LOD | ADD | PVE(%) |
| --- | --- | --- | --- | --- | --- | --- | --- |
| ***q2-1*** | CW | 2 | 0-3.5 | Marker7427-Marker159884 | 2.75 | 1336.51 | 4.81 |
|  | BD | 2 | 0-0.5 | Marker18813-Marker17835 | 4.68 | 6.56 | 6.36 |
|  | SH | 2 | 0-0.5 | Marker32141-Marker65230 | 7.06 | 31.82 | 6.89 |
| ***q2-2*** | CW | 2 | 19.5-23.5 | Marker61488-Marker48759 | 2.75 | 1261.92 | 3.99 |
| ***q2-3*** | DBH | 2 | 34.5-39.5 | Marker60282-Marker116898 | 3.43 | 0.32 | 13.45 |
| *q2-4* | SH | 2 | 92.5-94 | Marker91778-Marker70778 | 2.81 | 18.72 | 2.24 |
| *q3-1* | CW | 3 | 31.5-38.5 | Marker18298-Marker57278 | 2.64 | 1309.23 | 4.43 |
|  | SH | 3 | 31.5-36.5 | Marker18298-Marker140688 | 2.85 | 19.66 | 2.48 |
| *q3-2* | SH | 3 | 53.5-62.5 | Marker66024-Marker63134 | 2.82 | 18.72 | 2.23 |
| ***q4-1*** | BD | 4 | 82.5-83.5 | Marker15210-Marker16050 | 5.01 | 6.86 | 6.93 |
|  | SH | 4 | 82.5-83.5 | Marker9162-Marker6401 | 9.06 | 36.38 | 9.00 |
| *q4-2* | BD | 4 | 0-0.5 | Marker41190-Marker73435 | 2.93 | -5.14 | 3.98 |
| ***q6-2*** | CW | 6 | 60.5-62.5 | Marker54767-Marker135702 | 8.30 | 2372.95 | 14.88 |
|  | DBH | 6 | 57.5-58.5 | Marker109918-Marker44609 | 3.76 | 0.33 | 12.79 |
|  | BD | 6 | 60.5-62.5 | Marker54767-Marker135702 | 9.56 | 9.68 | 14.07 |
|  | SH | 6 | 60.5-62.5 | Marker54767-Marker135702 | 9.69 | 37.97 | 12.27 |
|  |  | 6 | 57.5-58.5 | Marker109918-Marker44609 | 2.79 | 7.28 | 0.92 |
| *q8-2* | BD | 8 | 57.5-64.5 | Marker36128-Marker3860 | 2.81 | 5.01 | 3.89 |
| *q9-1* | CW | 9 | 8.5-10.5 | Marker15339-Marker9283 | 2.56 | 1288.90 | 4.65 |
|  | BD | 9 | 8.5-10.5 | Marker15339-Marker57140 | 2.69 | 4.91 | 3.80 |
|  | SH | 9 | 8.5-11.5 | Marker15339-Marker29130 | 4.14 | 24.11 | 4.07 |
| *q9-2* | SH | 9 | 49.5-54.5 | Marker48199-Marker180320 | 2.76 | 19.27 | 2.34 |
| *q11* | SH | 11 | 86.5-89.5 | Marker51655-Marker59785 | 3.54 | 23.10 | 3.88 |
