# Supplementary material for: High-density genetic map construction and quantitative trait loci identification for growth traits in (Taxodium distichum var. distichum × T. mucronatum) × T. mucronatum
Source: BMC Plant Biol. 2018 Nov 1;18:263. doi: 10.1186/s12870-018-1493-0 (PMC6474422; doi:10.1186/s12870-018-1493-0)
Supplement: Supplementary file 8 — Result of the blast analysis of the markers within the intervals of the three stable QTLs against the unigene database of ‘Zhongshanshan’. (PDF 126 kb) [file 12870_2018_1493_MOESM9_ESM.pdf]

```
BLASTN 2.2.10 [Oct-19-2004]


Reference: Altschul, Stephen F., Thomas L. Madden, Alejandro A. Schaffer,
Jinghui Zhang, Zheng Zhang, Webb Miller, and David J. Lipman (1997),
"Gapped BLAST and PSI-BLAST: a new generation of protein database search
programs",  Nucleic Acids Res. 25:3389-3402.

Query= Marker20554
         (200 letters)

Database: Zhongshanshan 406.Unigene.fasta
           117,776 sequences; 77,714,018 total letters



                                                                 Score    E
Sequences producing significant alignments:                      (bits) Value

comp127245_c0                                                         174   3e-
043


>comp127245_c0
          Length = 2649

 Score =  174 bits (88), Expect = 3e-043
 Identities = 97/100 (97%)
 Strand = Plus / Plus


Query: 1    tgcacgtagatgctgagcctcctgtgttgcaccatgatgtcaaatctgccaatgttctgc 60
            |||||||||| |||||||||||||||||||||||||||||||||||||||||||||||||
Sbjct: 1940 tgcacgtagaagctgagcctcctgtgttgcaccatgatgtcaaatctgccaatgttctgc 1999


Query: 61   ttgtagacgacaaccatgccaaatttgcagattttggcct 100
            ||||||| ||||||||||||||| ||||||||||||||||
Sbjct: 2000 ttgtagatgacaaccatgccaaacttgcagattttggcct 2039



 Score =  167 bits (84), Expect = 7e-041
 Identities = 96/100 (96%)
 Strand = Plus / Plus


Query: 101  atgacaatgattgatccaacgcttaaagaagagcagattgatttggatgaattgaagttg 160
            |||||||| ||||||||||||||||| |||||||||||||||||||||||||||||||||
Sbjct: 2272 atgacaattattgatccaacgcttaatgaagagcagattgatttggatgaattgaagttg 2331


Query: 161  ataagaggggtattgaatgcctgtctgaataagaatggag 200
            |||| |||| ||||||||||||||||||||||||||||||
Sbjct: 2332 ataatagggatattgaatgcctgtctgaataagaatggag 2371
```

```
BLASTN 2.2.10 [Oct-19-2004]


Reference: Altschul, Stephen F., Thomas L. Madden, Alejandro A. Schaffer,
Jinghui Zhang, Zheng Zhang, Webb Miller, and David J. Lipman (1997),
"Gapped BLAST and PSI-BLAST: a new generation of protein database search
programs",  Nucleic Acids Res. 25:3389-3402.

Query= Marker39317
         (200 letters)

Database: Zhongshanshan 405.Unigene.fasta
           70,312 sequences; 67,915,621 total letters



                                                                 Score    E
Sequences producing significant alignments:                      (bits) Value

CL4975Contig1                                                         198   2e-
050


>CL4975Contig1
          Length = 1017

 Score =  198 bits (100), Expect = 2e-050
 Identities = 100/100 (100%)
 Strand = Plus / Minus


Query: 1   atgcactatcgacctgcttctcacccgacattttaaaatccaaacaagccaggcacatat 60
           ||||||||||||||||||||||||||||||||||||||||||||||||||||||||||||
Sbjct: 571 atgcactatcgacctgcttctcacccgacattttaaaatccaaacaagccaggcacatat 512


Query: 61  gatggttatacacattgaatatattccccttcctttgaa 100
           ||||||||||||||||||||||||||||||||||||||||
Sbjct: 511 gatggttatacacattgaatatattccccttcctttgaa 472



 Score =  198 bits (100), Expect = 2e-050
 Identities = 100/100 (100%)
 Strand = Plus / Minus


Query: 101 ccacattcaacttcagtatgctacattctacgggctttcgccactggcttaaacccaaaa 160
           ||||||||||||||||||||||||||||||||||||||||||||||||||||||||||||
Sbjct: 227 ccacattcaacttcagtatgctacattctacgggctttcgccactggcttaaacccaaaa 168


Query: 161 cacagacagggatgactgcatacagaggccatagagaaca 200
           ||||||||||||||||||||||||||||||||||||||||
Sbjct: 167 cacagacagggatgactgcatacagaggccatagagaaca 128
```

```
BLASTN 2.2.10 [Oct-19-2004]


Reference: Altschul, Stephen F., Thomas L. Madden, Alejandro A. Schaffer,
Jinghui Zhang, Zheng Zhang, Webb Miller, and David J. Lipman (1997),
"Gapped BLAST and PSI-BLAST: a new generation of protein database search
programs",  Nucleic Acids Res. 25:3389-3402.

Query= Marker29918
         (200 letters)

Database: Zhongshanshan 406.Unigene.fasta
           117,776 sequences; 77,714,018 total letters



                                                                 Score    E
Sequences producing significant alignments:                      (bits) Value

comp112880_c1                                                         174   3e-
043


>comp112880_c1
          Length = 972

 Score =  174 bits (88), Expect = 3e-043
 Identities = 98/100 (98%), Gaps = 1/100 (1%)
 Strand = Plus / Plus


Query: 1   gtcatacttacgcatttttccaaaacatcaaaatcttcattagcgatcatgaaatatca 60
           |||||||||| |||||||| ||||||||||||||||||||||||||||||||||||||||
Sbjct: 1   gtcatacttaggcatttt-ccaaaacatcaaaatcttcattagcgatcatgaaatatca 59


Query: 61  agttagcaagataagtgttgccataaatgacaacctaatg 100
           ||||||||||||||||||||||||||||||||||||||||
Sbjct: 60  agttagcaagataagtgttgccataaatgacaacctaatg 99
```
